# Corrosion mitigation characteristics of some novel organoselenium thiourea derivatives for acid pickling of C1018 steel via experimental and theoretical study

**DOI:** 10.1038/s41598-023-36222-0

**Published:** 2023-06-03

**Authors:** Hany M. Abd El-Lateef, Mai M. Khalaf, Mohamed Gouda, T. A. Yousef, Sayed H. Kenawy, Mortaga M. Abou-Krisha, Mohamed Alaasar, Saad Shaaban

**Affiliations:** 1 Department of Chemistry, College of Science, King Faisal University, 31982 Al-Hasa, Saudi Arabia; 2 Department of Chemistry, Faculty of Science, Sohag University, Sohag, 82524 Egypt; 3 College of Science, Chemistry Department, Imam Mohammad Ibn Saud Islamic University (IMSIU), 11623 Riyadh, Kingdom of Saudi Arabia; 4 Department of Toxic and Narcotic Drug, Forensic Medicine, Mansoura Laboratory, Medicolegal Organization, Ministry of Justice, Mansoura, Egypt; 5 Refractories, Ceramics and Building Materials Department, National Research Centre, El-Buhouth St., Dokki, Giza, 12622 Egypt; 6 Department of Chemistry, South Valley University, Qena, 83523 Egypt; 7 Institute of Chemistry, Martin Luther University Halle-Wittenberg, Halle, Germany; 8 Department of Chemistry, Faculty of Science, Cairo University, Giza, Egypt; 9 Department of Chemistry, Faculty of Science, Mansoura University, Mansoura, 35516 Egypt

**Keywords:** Materials science, Chemical engineering, Electrochemistry, Organic chemistry

## Abstract

Two organoselenium thiourea derivatives, 1-(4-(methylselanyl)phenyl)-3-phenylthiourea (**DS036**) and 1-(4-(benzylselanyl)phenyl)-3-phenylthiourea (**DS038**) were produced and categorized using FTIR and NMR (^1^H and ^13^C). The effectiveness of the above two compounds as C-steel corrosion inhibitors in molar HCl was evaluated using the potentiodynamic polarization (PD) and electrochemical impedance spectroscopy (EIS) techniques. PD findings indicate that **DS036** and **DS038** have mixed-type features. EIS results show that growing their dose not only changes the polarization resistance of C-steel from 18.53 to 363.64 and 463.15 Ω cm^2^ but also alters the double layer capacitance from 710.9 to 49.7 and 20.5 μF cm^−2^ in the occurrence of 1.0 mM of **DS036** and **DS038**, respectively. At a 1.0 mM dose, the organoselenium thiourea derivatives displayed the highest inhibition efficiency of 96.65% and 98.54%. The inhibitory molecule adsorption proceeded along the Langmuir isotherm on the steel substrate. The adsorption-free energy of the adsorption process was also intended and indicated a combined chemical and physical adsorption on the C-steel interface. FE-SEM studies support the adsorption and protective abilities of the OSe-based molecule inhibitor systems. In Silico calculations (DFT and MC simulations) explored the attraction between the studied organoselenium thiourea derivatives and corrosive solution anions on a Fe (110) surface. The obtained results show that these compounds can make a suitable preventing surface and control the corrosion rate.

## Introduction

Excellent mechanical qualities make carbon steel (C-steel) an essential material with a broad scope of uses in different areas including the marine and petroleum sectors^1^. C-steels are easily corroded in acidic environments, principally in hydrochloric acid that is used for industrial pickling, acid descaling, cleaning, and oil well acidizing^2^. The annual loss cost could be calculated to reach billions of dollars^3^. Although many strategies, including coating and deposition, were created to prevent metals from corroding^4–7^, the usage of corrosion inhibitors is still among the best and most efficient strategies^8,9^. Corrosion inhibitors are distinguished by their strong capacity for adhesion to metallic surfaces. The corrosion rate is immediately reduced when the inhibitor is added in modest amounts since it quickly prevents corrosion^10^.

Due to their abundance in adsorption centers, such as hetero atoms (sulfur, oxygen, and nitrogen), organic molecules are frequently utilized as efficient corrosion inhibitors for mild steel in aqueous conditions. This makes the inhibitors cost-effective^7,11,12^. The mutual contacts between the metal surface and the organic layer, which are controlled by an adsorption mechanism, can significantly slow down the rate of both anodic and cathodic corrosion reactions at the metal/solution interface^13,14^. While electrochemical techniques like electrochemical impedance spectroscopy and potentiodynamic polarization can measure the rate of corrosion, theoretical simulations can measure the interactions between metals and inhibitors^15^.

Organoselenium (OSe) hybrids have recently gained much attention as a result of their diverse applications, particularly in material and medicinal chemistry^16,17^. The selenium (Se) unprecedented characteristics and redox properties secured the potential biochemical and industrial applications of OSe agents^18^. The lower electronegativity and larger size of Se compared to its analogs sulfur, nitrogen, and phosphorous, are the main reasons for its higher polarizability and thus nucleophilicity^17^. Consequently, organoselenium (OSe) compounds are generally good nucleophiles and possess potential catalytic and chelating activities^17^. Unlike sulfur, Se is a semiconductor and showed photoconductive and photovoltaic properties it is therefore used extensively in material science and electronics, such as solar cells, sodium-ion batteries, photocells, and light meters^17^.

**Figure 1:**
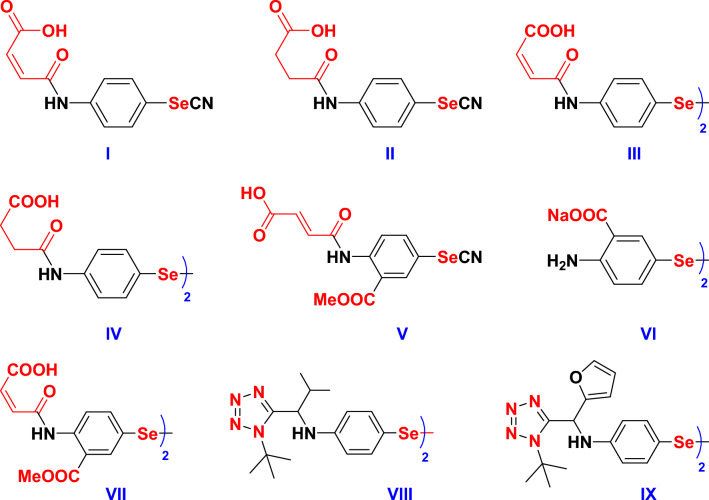
Organoselenium hybrids with promising corrosion inhibition properties.

During the past decades, many corrosion inhibitors were developed and have shown considerable inhibition activities; however, they only function well at room temperature and at low acid concentrations^8–10^. Therefore, inhibitors withstanding harsh conditions i.e., high temperature and concentrated acid (> 15 wt % HCl) are highly required in pipeline cleaning solutions and acidizing fluids, as well as in the petrochemical industries. Within this context, thioureas have shown potential application in retarding the corrosion of aluminum, copper, ferrous, zinc, and magnesium metals in different aggressive media by influencing the cathodic and anodic reactions. They acted as ideal adsorption sites in potential inhibitors owing to their ability to share free electrons with the metal template via their two nitrogen and sulfur atoms thus protecting the metals from acid corrosion by preventing the contact surface area available with hydrogen ions^16^. On the other hand, OSe compounds are considered better corrosion inhibitors than their organosulfur homologous owing to the Se greater ability to share its outer electrons with metals. Unfortunately, the anticorrosive efficacy of the OSe agents was seldom discussed and limited to a few examples in the literature. Recently, we reported different organoselenocyanates- (**I** and **II**) and diselenide-based (**III** and **IV**) water-soluble OSe compounds corrosion inhibitors for reinforced steel in the simulated concrete pore solution^19^. Moreover, we developed several OSe-tethered anthranilic acid hybrids (**V**, **VI**, and **VII**) as potential corrosion inhibitors for the J55 pipeline steel and 6061 aluminum alloy^20^. Additionally, we have also reported OSe-based tetrazoles (**VIII**, and **IX**) as promising corrosion inhibitors for the J55 steel tubing samples during oil well acidizing (Fig. 1)^21^.

It is therefore anticipated that a proper combination of thiourea and organoselenium compounds would enhance the overall corrosion inhibition activity. Accordingly, the present study focused on the preparation and structural investigation of organoselenium thiourea derivatives using a facile method, and spectroscopic methods (FTIR, ^1^H NMR, and ^13^C NMR). The study correspondingly involved the estimation of the synthesized OSe agents as inhibitors for C-steel corrosion in pickling solution using several electrochemical methods such as PDP and EIS. Furthermore, the molecular reactivity strictures on both the atomic and molecular levels were studied by means of DFT calculations to account for inhibitor–metal interactions, which is important to comprehend corrosion inhibition proficiency. The effectiveness of each inhibitor in relation to its orientation and structure, as well as the method by which an inhibitor adheres to metal surfaces, are thoroughly explained by the Monte Carlo simulations.

## Experimental work

### Materials, reagents, and methods

In this work, carbon steel with the following composition: P (0.046%), Si (0.35%), C (0.23%), Mn (1.42%), Cr (0.13%), Ni (0.034%), and Fe (rest) with an active area of ≈ 1 cm^2^ was used as the working electrode and scratched by various grades of emery papers (up to 1500 grade), degreased with acetone, rinsed with bidistilled H_2_O, and dried with faint tissues. The experiments were complemented in molar HCl solution in the nonappearance and existence of various doses of the examined organoselenium thiourea compounds **DS036** and **DS038**. For each test, the component concentrations were ordered from 2 × 10^–5^ to 1.0 × 10^–3^ M; a freshly discarded solution was used.

**Figure 2:**
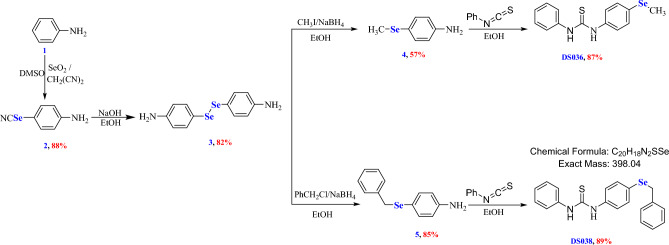
Synthesis of organoselenium compounds **2–5**, **DS036** and **DS038**. (**a**) Selenocyanate **2** was obtained in 88% yield from aniline (2.5 mmol), CH_2_(CN)_2_ (1.5 mmol), SeO_2_ (3 mmol), and DMSO (5 mL); (**b**) Diselenide **3** was synthesized in 82% yield from selenocyanate **2** (0.5 mmol), NaOH (0.5 mmol), and ethanol (6 mL); (**c**) Selenide **4** was obtained in 57% yield from diselenide **3** (1 mmol), methyl iodide (2.2 mmol), and EtOH (20 mL); (**d**) Selenide **5** was obtained in 85% yield from diselenide **3** (1 mmol), benzyl chloride (2.2 mmol), and EtOH (20 mL); (**e**) Thiourea **DS036** was obtained in 87% yield from selenide **4** (1 mmol), phenyl isothiocyanate (1.2 mmol), and EtOH (10 mL); (**f**) Thiourea **DS038** was obtained in 89% yield from selenide **5** (1 mmol), phenyl isothiocyanate (1.2 mmol), and EtOH (10 mL).

Melting points were recorded in degree centigrade using the Gallenkamp instrument. Spectra for the FTIR were recorded (KBr, ύ cm^−1^) at King Faisal University on Mattson 5000 FTIR Spectrophotometer. The ^1^H NMR and the ^13^C-NMR spectra were measured using Varian Spectrophotometer at 400 MHz and 500 MHz, employing the TMS internal reference and DMSO-*d*_6_ as the solvents. The chemical shifts (*δ*) in parts per million were recorded with respect to the residual peak of solvents. Compounds **2**–**5** were synthesized according to our literature reports (see Fig. 2) (see detailed experimental procedures in the Supporting Materials)^22–29^.

### Synthesis and characterization

The OSe thiourea derivatives **DS036** and **DS038** were efficiently synthesized from readily available chemicals (e.g., aniline, phenyl isothiocyanate, SeO_2_). All the reactions were straightforward and proceeded smoothly with classical and simple workups. The OSe thiourea derivatives **DS036** and **DS038** were obtained in moderate to high (57–89%) yields as well as in high purity as they recrystallized efficiently in ethanol without the need for sophisticated isolation using column chromatography. Furthermore, the **DS036** and **DS038** have different proton acceptor centers (e.g., nitrogen, sulfur, and Se) and this might be the reason for their good solubility in the HCl solution. Indeed, they are also readily soluble in different organic solvents such as acetone, DMF, and DMSO.

Interestingly, many organoselenium reactions were reported to proceed faster in water, and therefore the synthesis of **DS036** and **DS038** is worth further investigation under green conditions in the future once they are established as potential inhibitors.

#### General procedure for the synthesis of OSe thiourea derivatives DS036 and DS038.

To a solution of organic selenide **4** or **5** (1 mmol) in (10 ml) ethanol, phenyl isothiocyanate (1.2 mmol) was added, and the mixture was refluxed for 6 h. The formed precipitate was filtered and washed with ethanol and water. The thiourea derivatives were recrystallized from ethanol and were obtained in enough purity, and no further purifications were needed.

#### Synthesis of 1-(4-(methylselanyl)phenyl)-3-phenylthiourea (DS036)

Compound **DS036** was synthesized from methyl 4-(methylselanyl)aniline (**4**) (1 mmol, 187 mg) and phenyl isothiocyanate (1.2 mmol, 162 mg). The reaction was followed by TLC (EtOAc/hexane 1:2; *R*_f_ = 0.25) and isolated as a white solid with 87% yield (280.1 mg). Compound **DS036** was recrystallized from ethanol as white crystals and its MP was 161–162 °C. IR (FT‐IR, cm^−1^): 3199, 3025, 3001, 1685, 1599, 1522, 1449; ^1^H NMR (400 MHz, DMSO) δ 9.80 (d, *J* = 3.9 Hz, 2H, 2NH), 7.51 (d, *J* = 7.8 Hz, 2H, Ar–H), 7.46–7.33 (m, 6H, Ar–H), 7.15 (t, *J* = 7.3 Hz, 1H, Ar–H), 2.36 (s, 3H, SeCH_3_). ^13^C NMR (101 MHz, DMSO) δ 180.03, 139.90, 138.13, 130.37, 128.94, 127.24, 125.02, 124.95, 124.15, 7.38; MS (ESI): m/z = found 345.0 (M^+^ + Na), 321 (M^+^–H), 323 (M^+^ + H); calcd. 322.0

#### Synthesis of 1-(4-(benzylselanyl)phenyl)-3-phenylthiourea (DS038)

Compound **DS038** was synthesized from 4-(benzylselanyl)aniline (**5**) (1 mmol, 263 mg) and phenyl isothiocyanate (1.2 mmol, 162 mg). The reaction was followed by TLC (EtOAc/hexane 1:2; *R*_f_ = 0.25) and isolated as a white solid with 89% yield (354 mg). Compound **DS038** was recrystallized from ethanol as grey crystals and its MP was 156–158 °C. IR (FT‐IR, cm^−1^): 3198, 3022, 3000, 1599, 1588, 1522, 1449; ^1^H NMR (400 MHz, DMSO) δ 9.84 (s, 2H, 2NH), 7.52–7.41 (m, 6H, Ar–H), 7.35 (t, *J* = 7.8 Hz, 2H, Ar–H), 7.31–7.25 (m, 4H, Ar–H), 7.21 (td, *J* = 5.6, 2.4 Hz, 1H, Ar–H), 7.14 (dd, *J* = 15.9, 8.5 Hz, 1H, Ar–H), 4.22 (s, 2H, SeCH_2_). ^13^C NMR (101 MHz, DMSO) δ 179.92, 139.84, 139.25, 139.07, 132.86, 129.28, 128.95, 128.82, 127.24, 125.69, 124.98, 124.51, 124.14, 31.43; MS (ESI): m/z = found 421.0 (M^+^ + Na), 397 (M^+^-H), 399 (M^+^ + H); calcd. 398.04.

### Electrochemical corrosion inhibition measurements (EIS and PDP)

The carbon steel samples used in the EIS and PDP experimentations. All experimental runs were performed with the assistance of an electrochemical cell three-electrode system, which was connected to a Gamry potentiostat/galvanostat/ZRA (Reference 600+) electrochemical workstation at a 298 K. The working electrode was made of carbon steel, the counter electrodes were made of platinum sheet, and the reference electrode was made of silver/silver chloride (Ag/AgCl). EIS examination was achieved in a potentiostatic circumstance at a frequency range of 0.1 Hz to 100 kHz with a 10 mV AC signal amplitude and at open circuit potential (*E*_OCP_). The optimized EIS response was fitted using the Z-view software version 3.4. Following the EIS optimization, the PDP measurements were done under the same optimized conditions, where the potential was swept at a rate of 1.0 mV s^−1^ at *E*_OCP_, principally from ± 250.0 mV versus *E*_OCP_. The procedure of the LPR corrosion rate tests was similar to those defined in our previous work^10^. For each run of the experiment, three duplicate readings were recorded.

### DFT calculations and MC simulations

We used the DMOL3 program^30^ from the Materials Studio package^31^, which is intended for the implementation of large-scale density functional theory (DFT) computations, to perform cluster calculations. Calculations of DFT semi-core pseudopods (DSPP) were done using double numerical basis sets and polarization functional theory (DNP). The 6-31G Gaussian basis sets and the DNP basis sets are of equivalent quality^32,33^. Based on the generalized gradient approximation (GGA), the RPBE functional^34^ is currently the best exchange–correlation functional^35^ and is used to account for the effects of electron exchange and correlation. There were no symmetry restrictions when performing the geometric optimization.

By conducting Monte Carlo experiments on the exterior of Fe (110), the adsorption locator revealed the suitable adsorption formations of the protonated forms of the organoselenium base thiourea derivatives for MC simulations^36^. The particle energy of the thiourea derivatives was improved using the Forcite classical simulation engine^37^. This was done to calculate the ability of the organoselenium derivatives to inhibit. The organoselenium derivatives, HCl, and external Fe (110) were all adsorbed within a periodic boundary tuning in a simulated box with dimensions of 24.82°A × 24.82°A and 38.24°A. The universal simulation studies with a force field were operated to simulate the adsorption performance of organoselenium base thiourea derivatives on the surface of Fe (110).

## Results and discussions

### Synthesis of the Se-based thiourea derivatives

1-(4-(Methylselanyl)phenyl)-3-phenylthiourea (**DS036**) and 1-(4-(benzylselanyl)phenyl)-3-phenylthiourea (**DS038**) were prepared in good yields from the reaction of phenyl isothiocyanate with 4-(methylselanyl)aniline (**4**) and 4-(benzylselanyl)aniline (**5**), respectively (Fig. 2).

### Tafel curve measurements

**Figure 3:**
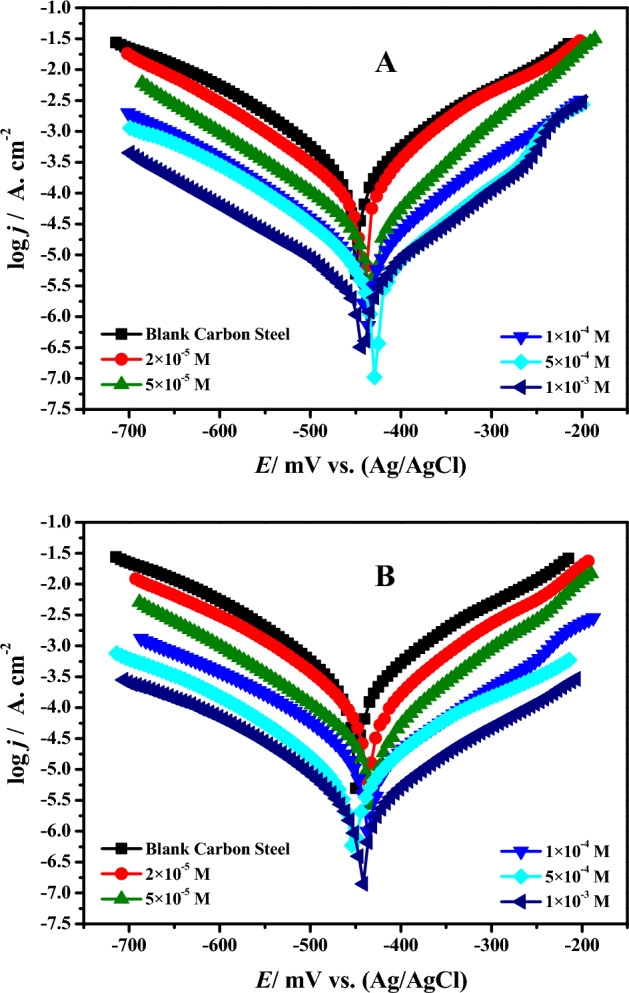
PDP plots for C-steel in molar HCl solution without and with different concentrations of OSe-based thiourea compounds (**a**) **DS036** and (**b**) **DS038**.

The corrosion current (*j*_cor_) considerably decreased after the corrosion inhibitors **DS036** and **DS038** were added, and the corrosion potential (*E*_cor_) likewise changed noticeably in either a negative or positive direction, as seen by the Tafel polarization plots in Fig. 3. In comparison to the uninhibited diagram, the form of the remaining anode area curve is extremely important in addition to the polarization curve with a **DS036** and **DS038** concentration of 0.002 mM. The polarization curve's slope quickly increases in the Tafel curve curves at concentrations of 1.0 mM. The shoulder in the polarization curve is associated with the adsorption complexity and desorption route of the active component in the medium and the hindering anode and cathode reaction mechanism^38^. The cathode region of all the polarization curves has a shape that is remarkably similar, which demonstrates that the addition of the OSe derivatives **DS036** and **DS038** has no effect on the reaction mechanism of dissolved oxygen reduction. As the **DS036** and **DS038** dose upsurges, the *j*_cor_ in the cathode area regularly declines, which designates that this barrier layer adsorbed by **DS036** and **DS038** on the C-steel surface could decrease the efficient center of the reduction of O_2_ on the metal interface, and with the upsurge in **DS036** and **DS038** dose, the development of a thicker protective layer will cause less active centers of oxygen reduction, resulting in inferior *j*_cor_.

The corrosion current of the C-steel electrode gradually decreased when the concentration of **DS036** and **DS038** is low (0.02 mM), indicating that only a small number of **DS036** and **DS038** compounds attach to the C-steel surface at this time. Although there is a slight corrosion inhibition effect, this is not acceptable to alter the reaction mechanism of the C-steel anode metal dissolution. This behavior matches the occurrence of a diffusion film on the C-steel surface in the EIS studies, which will be discussed later. When the **DS036** and **DS038** dose exceeds 0.50 mM, there are enough **DS036** and **DS038** molecules in the medium to form a denser protective coating on the surface of the C-steel, preventing the C-steel from deterioration.

The cathode and anode currents of all polarization curves containing **DS036** and **DS038** tend to drop, which shows that these molecules block their response to the cathode and anode regions of the C-steel surface. As a result, these molecules are mixed corrosion inhibitors for C-steel.

In order to reduce error when using the Tafel extrapolation method to fit the polarization curve data, the anode region of the polarization curve does not take part in the fitting. This is because the iron on the surface of the C-steel in the anode region will dissolve to a certain extent and the metal surface is relatively rough^39^. The *j*_cor_ values are fitted using the cathode region's polarization curve. The appropriate parameters are revealed in Table 1 which contains *E*_cor_, *j*_*c*or_, *β*_c,_ and *β*_a_ (cathodic and anodic Tafel slope), and the inhibition efficiency (*η*_i_/%) from the Tafel polarization plot is intended by the following Eq.^40^:

1 $$\eta_{i} / {\%} = \left( \frac{j_{cor,0} - j_{cor,i} }{j_{cor,0} } \right) \times 100$$

**Table 1:** PDP plots for C-steel in molar HCl solution without and with different concentrations of OSe-based thiourea compounds (**DS036** and **DS038**) at 298 K.

Inhibitor code	*C*_i_ (mol L^−1^)	*j*_cor_ (µA cm^−2^) ± SD	*E*_cor_ (mV) (SCE)	*β*_a_ (mV dec^−1^)	*− β*_*c*_ (mV dec^−1^)	*θ*	*μ*_*p*_%
Blank	0.0	821.7 ± 31.2	− 450.5	91.75	163.92	–	–
DS036	2.0 × 10^–5^	473.87 ± 24.3	− 440.6	84.13	173.66	0.4233	42.33
	5.0 × 10^–5^	321.61 ± 19.4	− 435.4	86.01	169.69	0.6086	60.86
	1.0 × 10^–4^	178.55 ± 11.5	− 434.8	81.07	174.87	0.7827	78.27
	5.0 × 10^–4^	68.28 ± 5.2	− 429.2	78.68	172.16	0.9169	91.69
	1.0 × 10^–3^	27.52 ± 2.1	− 442.8	85.93	175.71	0.9665	96.65
DS038	2.0 × 10^–5^	431.14 ± 22.6	− 438.4	82.27	176.87	0.4753	47.53
	5.0 × 10^–5^	297.04 ± 17.7	− 433.5	85.85	179.94	0.6385	63.85
	1.0 × 10^–4^	146.34 ± 9.8	− 437.6	90.01	180.62	0.8219	82.19
	5.0 × 10^–4^	41.33 ± 3.1	− 452.8	88.96	178.28	0.9497	94.97
	1.0 × 10^–3^	11.99 ± 1.5	− 439.4	88.48	176.18	0.9854	98.54

Amongst them, *j*_cor_,_0_, and *j*_cor_,_i_ are the corrosion current densities for C-steel electrodes in the blank without and with **DS036** and **DS038** molecules, respectively. As can be observed from Table 1, with the upsurge of **DS036** and **DS038** doses, the *j*_*c*or_ of C-steel is also decreased, and the *η*_i_/% is progressively enhanced. When the dose of **DS036** and **DS038** reached 0.002 mM, the *η*_i_/% of **DS036** and **DS038** was meaningfully improved, and the *η*_i_/% reached 42.33 and 47.53%, respectively. When the **DS036** and **DS038** dose reaches 1.0 mM, the *j*_cor_ are 27.52, and 11.99 μA/cm^2^, and the *η*_i_/% reaches 96.65, and 98.54%, respectively. The outcomes are significantly consistent with those of EIS.

### Impedance analysis

**Figure 4:**
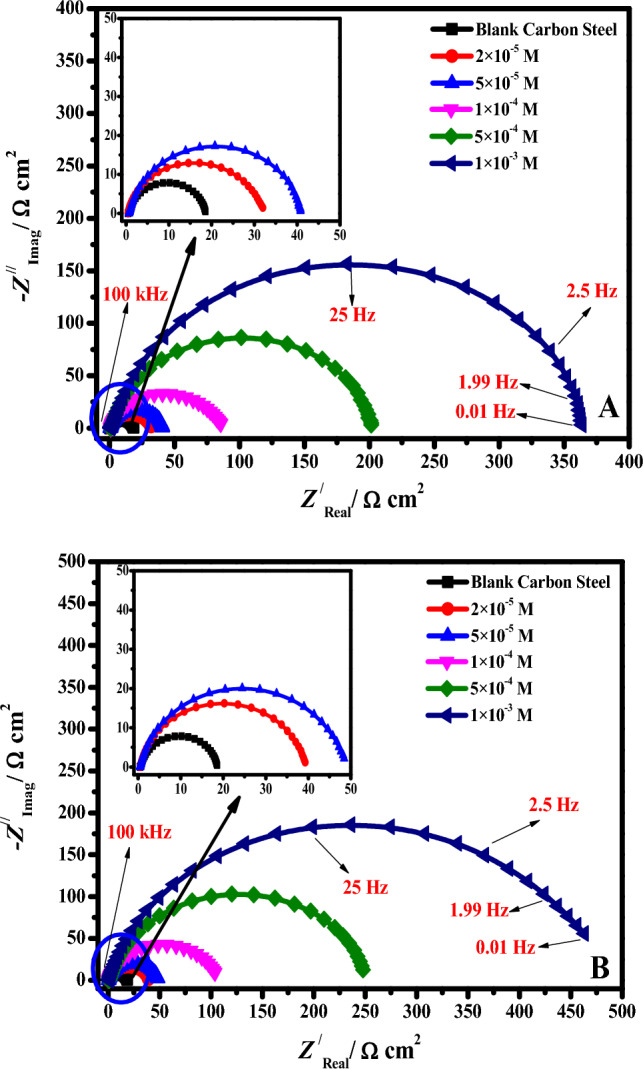
Nyquist plot of C-steel electrode in the blank molar HCl solution and with the addition of varying concentrations (**A**) **DS036** and (**B**) **DS038** at 298 K.

**Figure 5:**
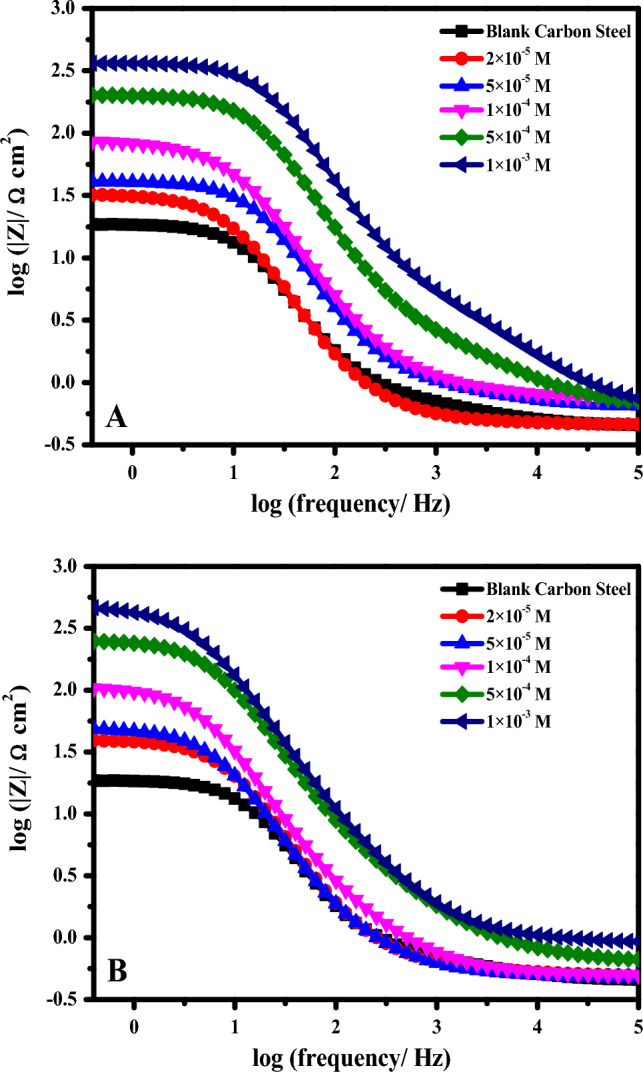
Bode plot of C-steel electrode in the blank molar HCl solution and with the addition of varying concentrations (**A**) **DS036** and (**B**) **DS038** at 298 K.

EIS was carried out to further examine the mechanism of **DS036** and **DS038** molecules' corrosion inhibition on C-steel. The OCP had reached a stable condition following 60 min of immersion of the C-steel specimen in molar HCl without and with various concentrations of **DS036** and **DS038** at 298 K. Based on this, the Nyquist and Bode plots for the C-steel electrode at a constant OCP were obtained and are shown in Figs. 4, 5. Figures 4, and 5 display the obtained EIS findings in Nyquist and Bode modules, respectively. This Figure clearly shows that all impedance spectra have a single capacitive loop, which demonstrates that the charge transfer process is primarily responsible for controlling the corrosion of C-steel in molar HCl with and without inhibitors and is regularly associated with the double-layer performance^41^. Additionally, these diagrams are identical for all concentrations tested, showing that the corrosion mechanism is unchanged^42^. The frequency dispersion of interfacial impedance may also be to blame for the fact that these Nyquist graphs are not perfect semicircles^43^. Chemical inhomogeneity, surface coarseness, and the adsorption–desorption process of inhibitive molecules on C- steel surfaces all contribute to this phenomenon^44^. Additionally, the semicircle’s diameter in the occurrence of the inhibitors **DS036** and **DS038** is larger than that observed in a molar HCl (blank solution) and grows with a growing inhibitor dose, which might be related to the expansion of the surface coverage of inhibitive compounds on the C-steel interface.

Figure 5 shows the appropriate Bode impedance modules for C-steel electrodes immersed in molar HCl with and without various inhibitor doses at its OCP. The increase of absolute impedance at low frequencies in the Bode plot confirms the higher protection with the increasing inhibitor concentration^43^. The concentration of **DS036** and **DS038** inhibitors in 1.0 M HCl is increased, as indicated by an increase in absolute impedance. This indicates superior inhibitive behavior because more inhibitor molecules are adsorbed on the C-steel surface at greater concentrations^45^.

**Figure 6:**
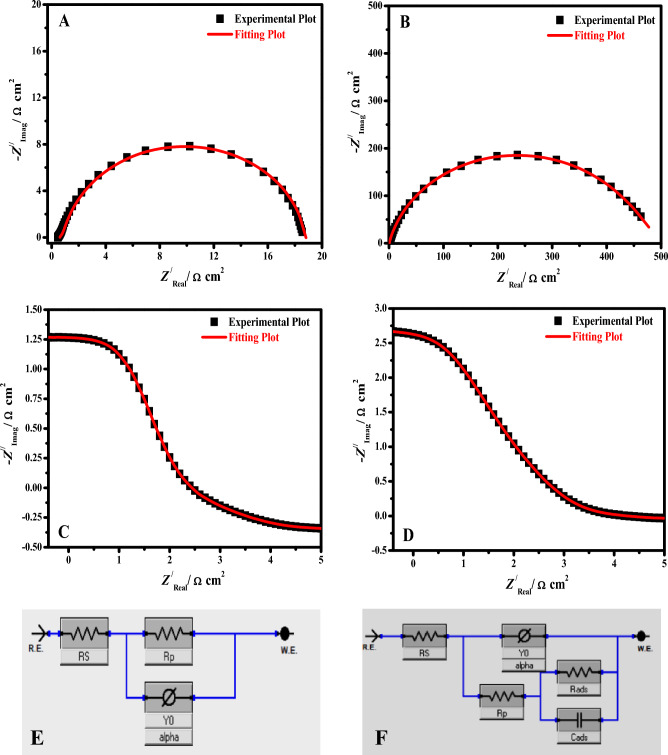
Comparison of Nyquist (**A**, **B**) and Bode diagrams (**C**, **D**) (black points) measured for steel specimens immersed in HCl and the simulated (red lines) for inhibited and uninhibited systems. EEC used in the fitting of data for the uninhibited electrode (**E**) and inhibited electrode (**F**).

Figure 6 shows the comparison of Nyquist (A, B) and Bode diagrams (C, D) (black points) measured for steel specimens immersed in HCl and the simulated (red lines) for inhibited and uninhibited systems. An electrochemical equivalent circuit (EEC) was used in the fitting for the data for uninhibited electrodes (E) and inhibited electrodes (F), where *R*_s_ is the electrolyte resistance, CPE (*Y*_0_, alpha) denotes a constant-phase element, and *R*_p_ denotes the polarization resistance (*R*_p_ = *R*_ct_ (charge transfer resistance) + *R*_f_ (film resistance)) in addition to the film capacitance (*C*_ads_), and inhibitor resistance (*R*_ads_) in the case of the inhibited system.

The element of CPE is utilized to clarify the capacitance semi-circle depression, which matches surface inhomogeneity resulting from impurities, surface coarseness, grain boundaries, displacements, additive adsorption, development of porous film, etc. The impedance CPE function is characterized by the following Eqn.^46^:

2 $$Z_{CPE} = \frac{1}{{Q(j\omega )^{\alpha } }}$$

where *j* represents the imaginary number (*j*^2^ = −1), Q signifies the CPE magnitude, and *ω* characterizes the angular frequency. *α* epitomizes the deviation restriction (−1 ≤ α ≤  + 1), which has the significance of a phase shift. The CPE denotes a pure resistor when *α* = 0, a pure capacitor when *α* =  + 1, and an inductor when *α* = −1^47^. Additionally, the following equation was used to get the double-layer capacitances, *C*_dl_, for a circuit that has a CPE^48^:

3 $$C{}_{dl} = Q(2\pi \omega_{\max } )^{\alpha - 1}$$

where *ω*_max_ is the maximum frequency value at the imaginary part of the EIS range. The electrochemical parameter values such as *n*, *R*_s_, *R*_p_, *C*_*dl*_, *Y*_0_, and ***η***_**E**_**/%** (inhibition capacity) of **DS036** and **DS038** were attained from EIS and recorded in Table 2. In the occurrence of DS036 and DS038, the values of *n* are found to be in the range of 0.792 to 0.884 and these values are more than the uninhibited solution (0.717). The higher *n* values in the inhibitor-containing systems exhibit that the DS036 and DS038 molecules produce a protective film over the C-steel surface and progress their homogeneity^10^. As the concentration of the inhibitor rises, the *R*_p_ values upsurge and the *C*_dl_ values decrease. The uppermost* R*_p_ (363.64 Ω cm^2^ for **DS036** and 463.15 Ω cm^2^ for **DS038**) has been found at an optimal dose (1.0 mM). The adsorption of inhibitors results in a rise in *R*_p_ values, suggesting a reduction in the exposed surface. However, a drop in *C*_dl_, which might be brought on by a reduction in the local dielectric constant and/or a rise in the thickness of the electrical double layer, suggests that the **DS036** and **DS038** inhibitors act by adsorption at the metal/medium interface. When the concentration of these OSe-based derivatives exceeds 1.0 mM, the values of inhibitory efficiency rise to 94.9% for **DS036** and 95.9% for **DS038**. These findings demonstrate once more that the prepared compounds have effective C-steel inhibitory performance in HCl solution and ***η***_**E**_**/%** follows the order: **DS038** > **DS036**. It is important to note that the inhibitory efficiencies determined by electrochemical measurements are essentially in agreement with those determined by PDP, as indicated in Table 1.

**Table 2:** EIS corrosion parameters of C-steel electrode in the blank molar HCl solution and with the addition of varying concentrations **DS036** and **DS038** at 298 K.

Inhibitor codes	*C*_inh*.*_ (mol/L)	*R*_s_ (Ω cm^2^)	*R*_P_ (Ω cm^2^) ± SD	*C*_*dl*_ (F cm^−2^) × 10^–6^	*Q*_CPE_		*χ*^2^ × 10^–4^	*θ*	*η*_E_ (%)
					*Y*_*0*_ (μΩ^−1^ s^n^ cm^−2^)	*n*			
Blank	0.0	0.45	18.53 ± 1.1	710.9	90.39	0.717	5.21	–	–
DS036	2.0 × 10^–5^	0.46	31.72 ± 3.5	285.7	56.84	0.792	4.28	0.415	41.5
	5.0 × 10^–5^	0.65	40.79 ± 4.2	230.9	40.34	0.814	4.29	0.545	54.5
	1.0 × 10^–4^	0.77	84.73 ± 6.5	129.7	29.64	0.856	5.16	0.781	78.1
	5.0 × 10^–4^	0.79	201.41 ± 12.4	55.5	11.86	0.835	5.25	0.908	90.8
	1.0 × 10^–3^	0.85	363.64 ± 18.8	49.7	4.52	0.856	5.18	0.949	94.9
DS038	2.0 × 10^–5^	0.49	39.29 ± 2.9	226.9	43.37	0.801	5.11	0.528	52.8
	5.0 × 10^–5^	0.57	48.42 ± 4.6	187.4	31.12	0.818	3.79	0.617	61.7
	1.0 × 10^–4^	0.69	103.73 ± 8.7	117.1	22.86	0.844	4.97	0.821	82.1
	5.0 × 10^–4^	0.76	247.91 ± 16.8	29.7	9.14	0.860	4.83	0.925	92.5
	1.0 × 10^–3^	0.98	463.15 ± 24.2	20.5	3.44	0.884	5.14	0.959	95.9

### Effect of temperature

**Figure 7:**
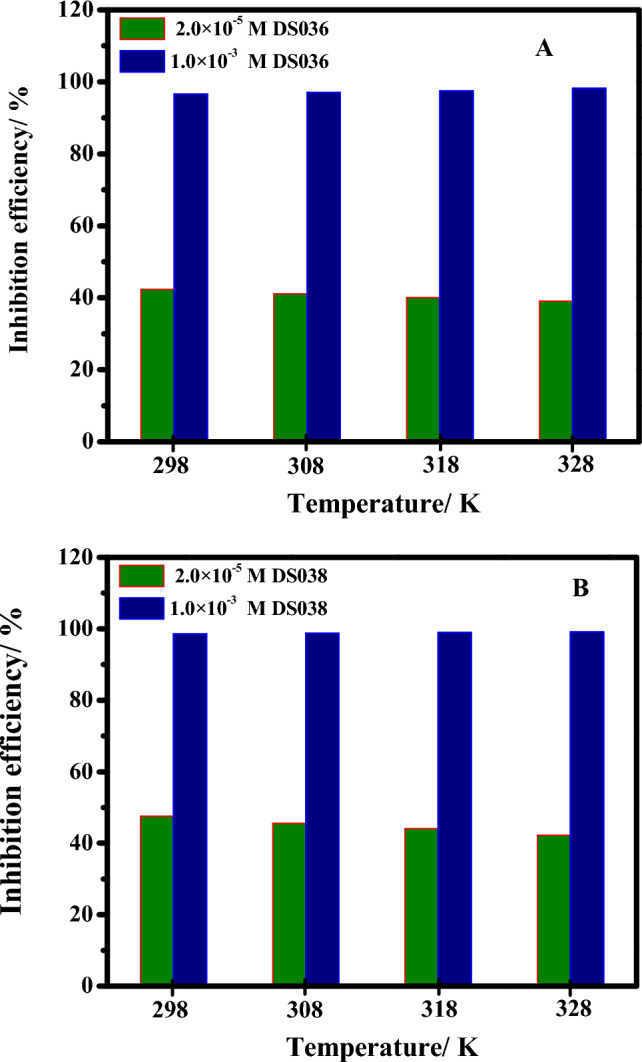
Relationship between inhibition efficiency and temperature in (**A**) DS036 and (**B**) DS038 at different concentrations.

The effect of temperature on C-steel corrosion in an HCl medium in the absence and existence of different inhibitor doses has been examined at the temperature range (298 − 328 K) using the EIS method. Figure 7 shows the relationship between inhibition efficiency and temperature in (A) DS036 and (B) DS038 at different concentrations. The inhibition efficiency for DS036 was changed from 42.3, and 96.6% at 298 K to 39.1, and 98.2% at 328 K, in the presence of 2.0 × 10^−5^ and 1.0 × 10^−3^ M, respectively (Fig. 7A). While, in the presence of the DS038 compound, the inhibition efficiency was altered from 47.5, and 98.54% at 298 K to 42.2, and 99.1% at 328 K in the presence of 2.0 × 10^−5^ and 1.0 × 10^−3^ M, respectively, respectively (Fig. 7B). Consequently, DS036 and DS038 inhibitor systems still display remaining protection performance to protect C-steel from corrosion by producing an adsorption layer on the metal interface even at higher temperature^21^. By comparing the activation energy (*E*_a_) in the presence and absence of the corrosion inhibitor, some important details concerning the adsorption mechanism of the inhibitor can be learned. The *E*_a_ values in the presence of organoselenium thiourea derivatives are seen to increase as the inhibitor concentration increases. The data demonstrate that the investigated electrode's *E*_a_ values in the analyzed corrosive media in the presence of DS036 and DS038 (35.25 and 40.65 for DS036 and DS038 respectively) are greater than those in the uninhibited medium (12.97 kJ/mol). As a result, the presence of an inhibitor raises the activation energy barrier of the tested electrodes' corrosion and hence lowers the corrosion rate. We can draw the conclusion that the increase in corrosion activation energy in the presence of DS036 and DS038 is commonly attributed to the development of a physical-character protective adsorption layer. The inhibitor system is physically adsorbed at lower temperatures, whereas chemisorption is more advantageous as the temperature rises^21^.

### Effect of immersion time

**Figure 8:**
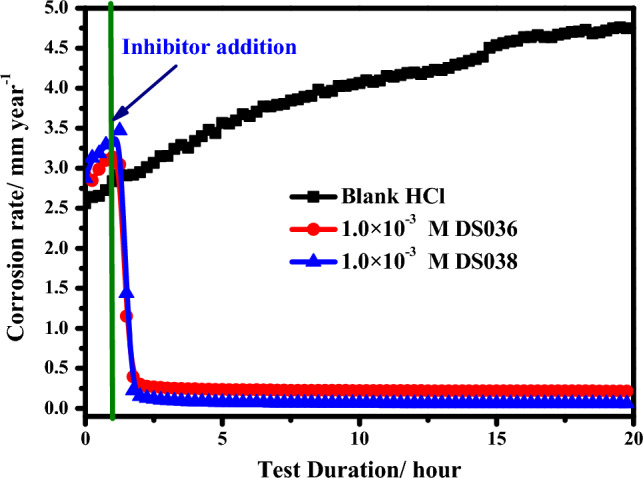
Variant of the corrosion rate with duration time for C-steel in 1.0 M blank HCl and containing 1.0 × 10^–3^ M of DS036 and DS038 at 25 °C.

In order to examine the stability of the adsorptive inhibitor film with time, the influence of exposure time on the corrosion rate was studied. The change of the corrosion rate with duration time for C-steel in 1.0 M blank HCl and containing 1.0 × 10^–3^ M of DS036 and DS038 at 25 °C was presented in Fig. 8. After one hour of immersion in the corrosive solution, the organoselenium thiourea compounds were introduced because at this point the *E*_cor_ became stable. CR was discovered to range between 2.62 and 4.76 mm/year in the blank medium. The CR of C-steel steadily increases with immersion duration in the blank media, as is shown in Fig. 8. The CR of C-steel was clearly expressively reduced by the addition of the synthetic organoselenium thiourea compounds. The CR reduced from 4.62 to 0.21 and 0.06 mm/year in the presence of 1.0 × 10^–3^ M of DS036 and DS038, respectively, after 20 h of immersion. It might be accredited to its adsorption and formation of a protective layer on the metallic surface. During 2 to 20 h of exposure, the CR in DS036 and DS038-inhibited HCl is practically constant, showing that the adsorption film for DS036 and DS038 is stable and does not rupture with time.

### Adsorption isotherm studies

**Figure 9:**
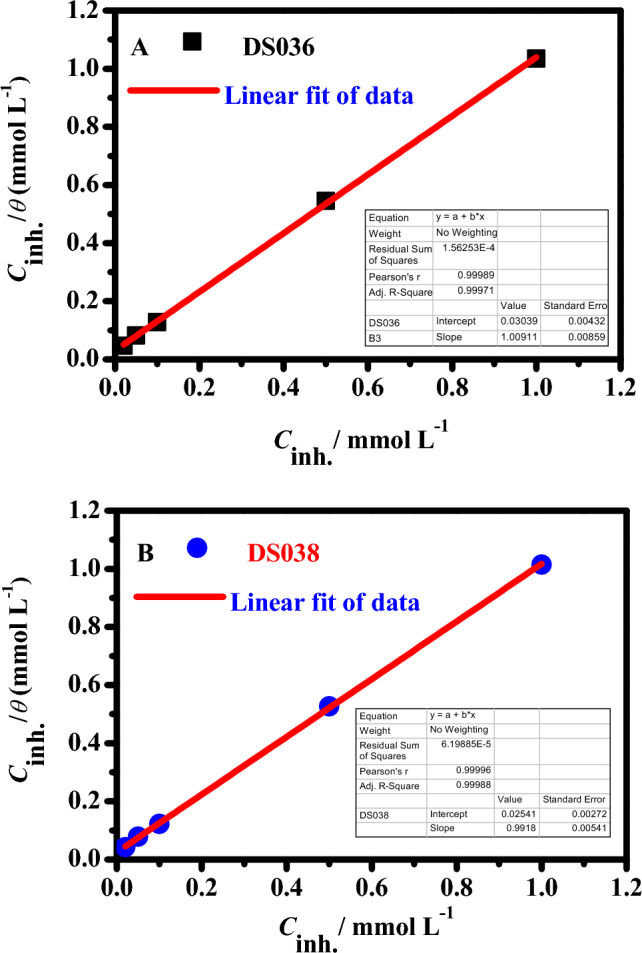
Adsorption model of Langmuir isotherm of inhibitors (**DS036**, and **DS038**) on the C-steel surface in molar HCl at 298 K.

Assuming that the inhibitory effect is primarily caused by adsorption at the metal/solution contact will allow us to determine the adsorption isotherm. The adsorption isotherm can give fundamental details on the adsorption of inhibitors on the metal surface. The fractional surface coverage values (*θ*) as a function of inhibitor concentration must be ascertained in order to derive the isotherm. The surface coverage (*θ*) values could be simply measured from the PDP studies by the ratio inhibition efficiency/100. Therefore, empirical research is required to establish which isotherm best describes the adsorption of inhibitors on the surface of C-steel. Numerous adsorption models such as Langmuir, Frumkin, Freundlich, and Temkin isotherms were characterized. Among the different models of adsorption isotherms tried, the most appropriate one was selected with the help of the correlation coefficient (*R*^2^) (Table S1). Langmuir isotherm was found to be the most fitting to the experimental findings, with all correlation coefficient values very close to unity, confirming that the adsorption process of OSe-based compounds on C-Steel in 1.0 M HCl follows Langmuir isotherm model. The isotherm model of Langmuir is described by the following Eqn.^49^:

4 $$\left( {\frac{{C_{inh} }}{\theta }} \right) = \left( {\frac{1}{{K_{ads} }}} \right) + C_{inh}$$

where *K*_ads_ and *C*_inh_ are the adsorption equilibrium constant and the inhibitor molar concentration. Figure 9 shows a straight line in the plot of the log (*C*_inh_*/θ* vs. *C*_inh_). The linear regression coefficients (*R*^2^) are nearly equivalent to 0.999 for **DS036**, and **DS038** inhibitors, which indicates that the examined compounds adhere to Langmuir's adsorption isotherm during adsorption in 1.0 M HCl solution. The adsorption-free energy ($\Delta G_{ads}^{0}$) is interrelated to the *K*_ads_ with the following Eqn.^50,51^:

5 $$\Delta G_{ads}^{0} = - RT\ln (55.5K_{ads} )$$

where the value 55.5 is the molar concentration of H_2_O. The values of *K*_ads_ and $\Delta G_{ads}^{0}$ were calculated at 298 K and. The *K*_ads_ values were found to be 9.91 × 10^3^ and 10.02 × 10^3^ M^−1^ for **DS036**, and **DS038** inhibitors, respectively. Moreover, the $\Delta G_{ads}^{0}$ values were found to be − 32.74 and − 32.81 kJ mol^−1^ for **DS036,** and **DS038** inhibitors, The instability of the adsorbed layer on the C-steel surface and the spontaneity of the adsorption process were both established by the negative values of $\Delta G_{ads}^{0}$. Additionally, the **DS038** inhibitor's high $\Delta G_{ads}^{0}$ value demonstrated that it is more strongly adsorbed on the C-steel surface in 1.0 M HCl than the **DS036** molecule. This is well-aligned with the range of inhibitory efficiency values discovered using electrochemical methods. It is common knowledge that values of $\Delta G_{ads}^{0}$ the order of − 20 kJ mol^−1^ or below suggest physisorption, while values more negative than − 40 kJ mol^−1^ include the sharing or electron transfer from the inhibitor molecules to the steel surface to form a coordinate type of bond (chemisorption). In this report, the $\Delta G_{ads}^{0}$ values ranging between − 20 and − 40 kJ mol^−1^ obviously specify its combined chemical and physical adsorption on the C-steel interface^52^.

### Surface morphology by FE-SEM

**Figure 10:**
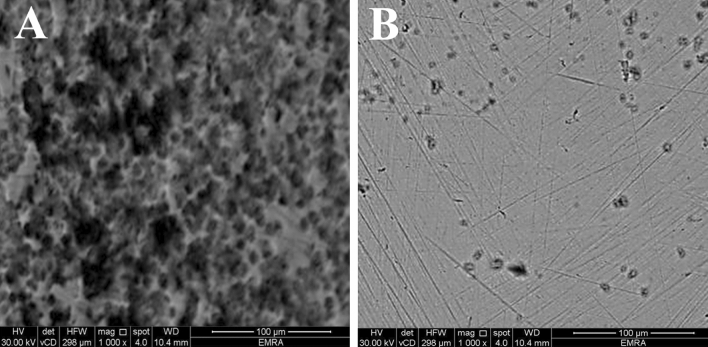
FE-SEM pictures for C-steel in blank 1.0 M HCl (**A**) and containing 1.0 × 10^−3^ M DS038 compound after 20 h of immersion.

FE-SEM analysis of C-steel conducted after 20 h of exposure to blank HCl (A) and 1.0 103 M DS038 is shown in Fig. 10A, B. The metal surface was severely corroded and degraded, with some pits and deep cavities, as evidenced by close examinations of the FE-SEM image acquired in the absence of the DS038 inhibitor (Fig. 10A). The metal sample has a superior morphology and smooth interface compared to the C-steel surface immersed in the blank medium when DS038 inhibitor is present (Fig. 10B). According to this, using DS038 inhibitor slows down the corrosion rate by preventing C-steel dissolving. This indicates effective corrosion inhibition.

### DFT studies

**Figure 11:**
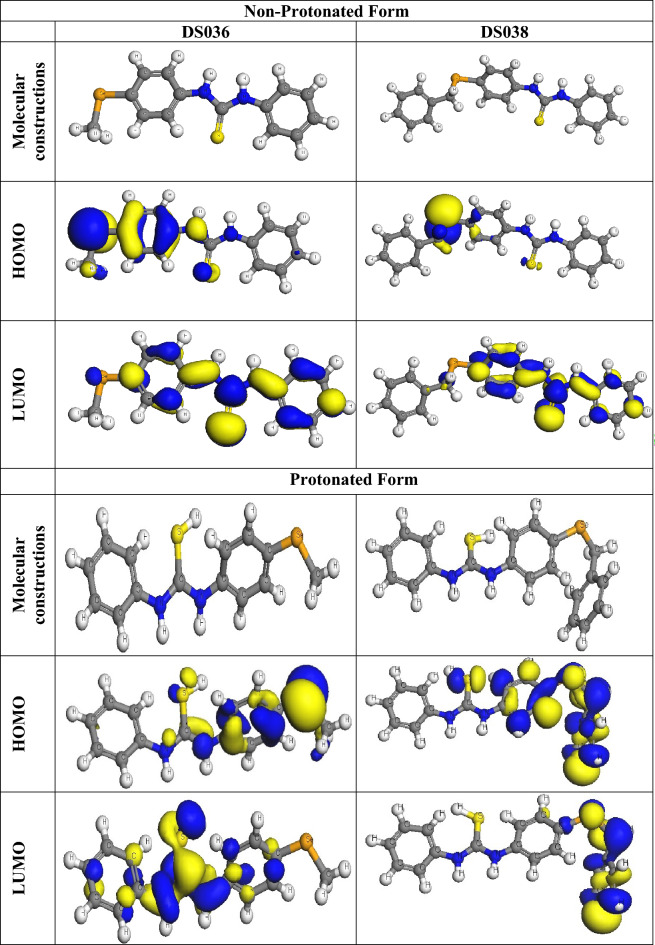
The optimized molecular constructions, HOMO, and LUMO for **DS036** and **DS038**.

Figure 11 shows the optimized structures, including the HOMO and LUMO distributions for organoselenium base thiourea derivatives. Table 3 presents the corresponding quantum chemical descriptors. According to frontier orbital theory, the HOMO and LUMO energies^53,54^ explain the donor or acceptor interactions at the interface between the inhibitor molecule and metal exterior. Consequently, high and low readings for *E*_HOMO_ and *E*_LUMO_, respectively, point to a corrosion prohibition that is strengthened by the presence of the compounds under investigation. In Table 3, DS038 has a higher HOMO value of − 4.56 eV when compared with DS036 (− 5.33 eV) while in protonated form were 5.06 and 5.19 eV for DS038 and DS036 respectively. As shown in Fig. 11, the HOMO levels for **DS036** were located at the seloxy, thiole, imine, and one of the phenyl rings, suggesting that the nitrogen, Se, and sulfur atoms were the favored positions for electrophilic attacks on the metal exterior. The HOMO levels in **DS038** were found at the seloxy, thiole, and imine positions, implying that the nitrogen, Se, and sulfur atoms were the preferred positions for electrophilic attacks on the metal exterior. This could increase the potency of the prohibition caused by the **DS036** and **DS038** derivatives' adsorption on the steel surface. In contrast, the *E*_LUMO_ value was − 1.61 eV for **DS038** (Table 3), which was less than − 1.66 eV for **DS036**. We observe that small differences in values between the inhibitors demonstrate the inhibitors' low inhibition activity differences, where better inhibition efficiency was observed for **DS038**. These results show that protonated species have a stronger propensity than non-protonated molecules to adsorb on the surface of the C-steel.

**Table 3:** DFT parameters of **DS036** and **DS038** compounds.

Compound		*E*_*HOMO*_ (eV)	*E*_*LUMO*_ (eV)	*∆E* (eV)	*IE*	*EA*	*η*	*σ*	*χ*	*∆N*	*μ*
Non-Protonated Form	**DS036**	− 5.33	− 1.66	3.67	5.33	1.66	1.84	0.54	3.50	0.36	3.51
	**DS038**	− 4.56	− 1.61	2.75	4.56	1.61	1.48	0.68	3.09	0.60	4.15
Protonated Form	**DS036**	− 5.19	− 4.35	0.84	5.19	4.35	2.38	0.42	4.77	0.06	3.71
	**DS038**	− 5.06	− 4.37	0.69	5.06	4.37	2.89	0.35	4.72	0.14	4.48

Important parameters, including the energy gap (∆*E*), HOMO, LUMO, global hardness (*η*), softness (*σ*), electronegativity (χ), and electron transfer fraction (∆*N*) were determined from the DFT calculation from the Eqns. 6–11^38,55^.

6 $$IE = - E_{{{\text{HOMO}}}}$$

7 $$EA = - E_{{{\text{LUMO}}}}$$

8 $$\eta = \frac{1}{2}(IE - EA) = \frac{1}{2}(E_{LUMO} - E_{HOMO} )$$

9 $$\chi = \frac{1}{2}(IE + EA) = \frac{1}{2}( - E_{LUMO} - E_{HOMO} )$$

10 $$\sigma = \frac{1}{\eta }$$

11 $$\Delta N = \frac{{\chi_{Fe} - \chi_{inh.} }}{{2(\eta_{inh.} + \eta_{Fe} )}}$$

where the electronegativity was 4.82 eV for Fe has been applied for the calculation of electron transfer (∆*N*).

The energy gap (∆*E*) is a crucial component in enhancing the organoselenium derivatives' ability to suppress corrosion. Better inhibitory potencies were suggested by lower ∆E values^56^. Table 3 shows that **DS038** has a lower ∆*E* value than **DS036** (2.75 eV) and 3.67 eV. This suggested a greater propensity for **DS038** adsorption on the outside of the C-steel. The lower values of electronegativity (*χ*) for **DS038** revealed that DS038 had a greater capacity to supply electrons to the metal than **DS036**^57^. A compound's stability and reactivity can be used to determine how hard or soft it is. During the adsorption process, soft chemicals, which are more reactive than hard ones, easily supply electrons to a C-steel sample. They function effectively as corrosion inhibitors as a result^58^. The **DS038** has a greater value (0.68), while the **DS036** has a higher value, as indicated in Table 3. (1.84). This demonstrated **DS038** simplicity in supplying electrons to the tested substance. As a result, high potency emerged.

The ∆N values demonstrated a particle's propensity to give electrons to the surface. A high ∆N value indicates that an inhibitor has a greater capacity for electron donation. When ∆*N* 3.6 is present, the strong electron-contributing ability improves the prohibition performance^59^. The determined values of N are shown in Table 3. The fact that **DS038's** ∆*N* value was larger (0.60) than **DS036's** (0.36) suggested that **DS038** was more likely to contribute electrons to the sample under study.

A powerful aspect that supports corrosion restriction is the dipole moment^60^. An improvement in the deformation energy and an improvement in the adsorption of an inhibitor on the steel surface are both indicated by an increase in the dipole moment. Therefore, an increase in the dipole moment will boost the effectiveness of the prohibition^61^. Table 3 demonstrates that **DS038** has a greater dipole moment (4.15 Debye) than **DS036** (3.51 Debye) while 4.48, 3.71 Debye for **DS038** and **DS036** in the case of protonated form. This demonstrated the increased propensity of **DS038** to adsorb on the C-steel surface, resulting in increased prohibition potency. Indicating that dipole–dipole interactions predominate more in the interaction between the protonated form and the C-steel interface than in the interaction between the C-steel surface and the non-protonated form, the values are higher for the protonated molecules than for the non-protonated molecules. It can be determined that the protonated organoselenium base thiourea derivatives forms are more effective at resisting corrosion than the non-protonated organoselenium base thiourea derivatives forms based on the above-calculated quantum characteristics.

### MC simulations

**Figure 12:**
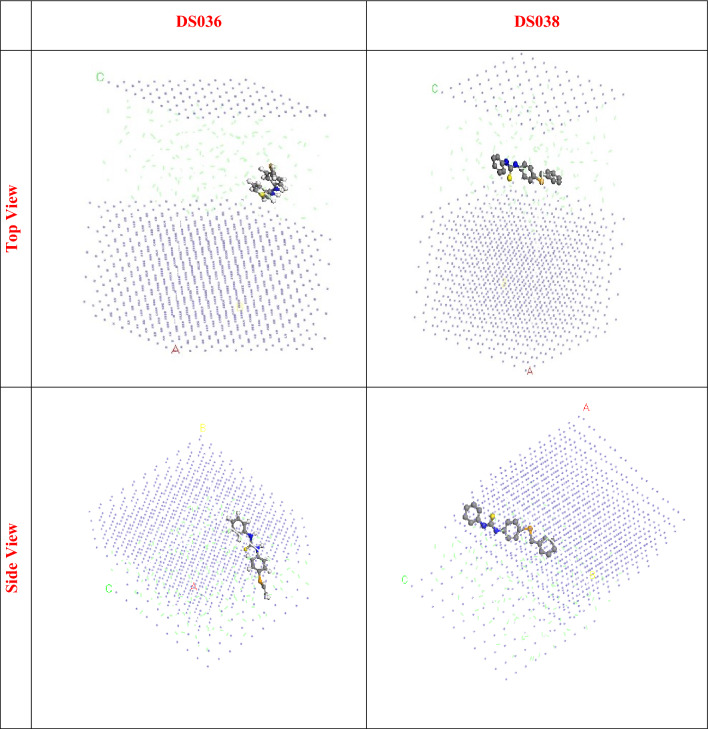
The adsorption locator module achieved the maximal suitable conformation for the adsorption of **DS036** and **DS038** on Fe (110) substrate.

The interactions of the organoselenium base thiourea derivatives with the C-steel surface and the mechanism of adsorption were visualized using MC simulations. The most plausible organoselenium derivative adsorption arrangements on the steel sample are depicted in Fig. 12. The adsorption locator module, which displays the smooth disposition and recommends an improvement in the adsorption with the largest surface coverage, was responsible for achieving this. In addition, Table 4 compiles the results of the Monte Carlo simulations. Table 4 provides the stiff adsorption energies for relaxed adsorbate compounds, relaxed adsorbate compound deformation energies, and relaxed adsorbate compound adsorption energies^62–67^. Compared to **DS036** the **DS038** displayed a greater negative value for adsorption energy (− 513.41 kcal mol^−1^) (− 407.22 kcal mol^−1^). This demonstrated that **DS038** adhered to the C-steel surface more strongly than **DS036** did, forming a stable adsorbed barrier that inhibits C-steel corrosion and indicating a stronger prohibition tendency for **DS038** than for **DS036**.

**Table 4:** Data and descriptors obtained using the Monte Carlo simulation for the adsorption of **DS036,** and **DS038** compounds on Fe (110).

Compound		Total energy	Adsorption energy	Rigid adsorption energy	Deformation energy	Inhibitor: d*E*_ads_/d*N*_i_	HCl: d*E*_ads_/d*N*_i_
Non-Protonated Form	**DS036**	− 362.61	− 407.22	− 407.70	− 487.70	− 44.78	− 2.53
	**DS038**	− 359.08	− 513.41	− 416.29	− 497.12	− 55.42	− 3.06
Protonated Form	**DS036**	− 258.59	− 408.54	− 308.92	0.3755	− 36.76	− 2.02
	**DS038**	− 227.31	− 583.34	− 283.63	0.2872	− 28.27	− 2.49

When the energy of the adsorbate was ignored, the d*E*_ads_/d*N*_i_ figures helped to understand the energy of the metal adsorbate configuration. The higher d*E*_ads_/d*N*_i_ values for **DS038** (55.42 kcal mol^−1^), as opposed to DS036 (44.78 kcal mol^−1^), show that **DS038** had larger adsorption than **DS036**. A further indication that **DS038** and **DS036** have a higher affinity for adsorption than HCl is the fact that HCl molecules have a d*E*_ads_/d*N*_i_ ratio of − 3.06 kcal mol^−1^. Thus, **DS038** and **DS036** formed a reliable barrier of protection and were irrevocably adsorbed on the Fe surface. The d*E*_ads_/d*N*_i_ figures help to clarify the energy of the metal adsorbate arrangement when the energy of the adsorbate was ignored. **DS038** had greater adsorption than **DS036**, as evidenced by the higher dEads/dNi values for **DS038** (− 55.42 kcal mol^−1^) compared to **DS036** (− 44.78 kcal mol^−1^). In addition, the fact that HCl molecules have a d*E*_ads_/d*N*_i_ value of − 3.06 kcal mol^−1^ indicates that **DS038** and **DS036** have a larger affinity for adsorption than HCl. Thus, **DS038** and **DS036** created a solid protective barrier and were definitively adsorbed on the Fe surface. It's interesting to note that these results match the above DFT and empirical results.

## Conclusions

OSe-based derivatives were synthesized and their mitigation performance for C- steel in an acidic pickling solution was inspected from both theoretical and experimental characteristics. The obtained results show that these compounds can make a suitable preventing surface and control the corrosion rate. The inhibition efficiencies are 96.65% and 98.54% in the presence of 1.0 mM of **DS036** and **DS038**, respectively. The synthesized OSe-based compounds were mixed-type inhibitors, according to the PD data, and the inhibition efficiency increased with increasing inhibitor doses. The highest *R*_p_ (363.64 Ω cm^2^ for **DS036** and 463.15 Ω cm^2^ for **DS038**) have been found at an optimal dose (1.0 mM). These molecules combined chemisorption and physisorption adsorption onto the surface of the C-steel and followed the Langmuir adsorption isotherms. The effects of immersion time and temperature reinforced further the performance of both synthesized OSe-based compounds. The FE-SEM analysis revealed a smoother electrode surface after the addition of the OSe-based molecules, proving that a shielding film had been formed to shield the steel substrate from contact with aggressive ions. The outcomes of the MC simulations and DFT calculations were in good agreement with the experimental findings.

## Supplementary Information

Supplementary Information.

## Data Availability

All data generated or analyzed during this study are included in this published article and its supplementary information files.
