# Supplementary material for: Corrosion mitigation characteristics of some novel organoselenium thiourea derivatives for acid pickling of C1018 steel via experimental and theoretical study
Source: Sci Rep. 2023 Jun 3;13:9058. doi: 10.1038/s41598-023-36222-0 (PMC10239482; doi:10.1038/s41598-023-36222-0)
Supplement: Supplementary file 1 — Supplementary Information. [file 41598_2023_36222_MOESM1_ESM.pdf]

Supporting Information

Corrosion mitigation characteristics of some novel organoselenium thiourea derivatives for acid pickling of C1018 steel via experimental and theoretical study

Hany M. Abd El-Lateef [1,2*], Mai M. Khalaf [1,2,], Mohamed Gouda[1,], T. A. Yousef [3,4], Sayed H. Kenawy[3,5] , Mortaga M. Abou-Krisha[3,6], Mohamed Alaasar[7,8] and Saad Shaaban[1,9*]

[1] Department of Chemistry, College of Science, King Faisal University, Al-Ahsa 31982, Saudi Arabia

[2] Department of Chemistry, Faculty of Science, Sohag University, Sohag 82524, Egypt

[3]College of Science, Chemistry Department, Imam Mohammad Ibn Saud Islamic University (IMSIU), Riyadh 11623, KSA

[4]Department of Toxic and Narcotic drug, Forensic Medicine, Mansoura Laboratory, Medicolegal organization, Ministry of Justice, Egypt

[5]Refractories, Ceramics and Building Materials Department, National Research Centre, El-Buhouth St., Dokki - 12622, Giza, Egypt

[6]Department of Chemistry, South Valley University, Qena, 83523, Egypt

[7]Institute of Chemistry, Martin Luther University Halle-Wittenberg, Halle (Saale), Germany Germany

[8]Department of Chemistry, Faculty of Science, Cairo University, Giza, Egypt

[9]Department of Chemistry, Faculty of Science, Mansoura University, 35516 Mansoura, Egypt

***** Correspondence: **hmahmed@kfu.edu.sa, hany_shubra@science.sohag.edu.eg** (H M Abd El-Lateef), **sibrahim@kfu.edu.sa** (S. Shaaban)

Synthesis of 4-selenocyanatoaniline (2)

Selenium dioxide (6 mmol) was added under stirring to a solution of malononitrile (3 mmol) in DMSO (15 ml). The mixture was stirred at room temperature for 15 min to obtain tri-selenium dicyanide. When the exothermic reaction had finished aniline (5 mmol) was added. The mixture was stirred for 20 min. Water (150 ml) was added to the reaction mixture and the resulting precipitate (4-selenocyanatobenzenamine) was filtered off, dried, and used without further purifications.4-Selenocyanatoaniline (**2**) was synthesized a yellow solid (88% yield) according to our reported literature method [24-28], mp: 73–74 °C. ^{1}H NM`R (400 MHz, $CDCl_3$) δ 7.44 (d, J = 8.4 Hz, 2H, Ar-H), 6.64 (d, J = 8.4 Hz, 2H, Ar-H), 3.95 (s, 2H, NH_2).

Synthesis of 4-(2-(4-aminophenyl)diselanyl)benzenamine (3)

Under argon, $NaBH_4$ (3 mmol) was added in small portions with caution to a solution of 4-selenocyanatobenzenamine (1 mmol) in absolute ethanol (40 ml). The mixture was stirred at room temperature for 2 h. The solvent was removed under reduced pressure and the remaining residue was dissolved in dichloromethane, and washed with water (3x50 ml). The organic layer was separated, dried with anhydrous Na_2SO_4, and removed under vacuum. The residue was purified by chromatography on silica gel (petroleum ether: ethyl acetate 4:2). 4,4'-diselanediyldianiline (**3**) was synthesized as pale-yellow crystals (82% yield) according to our reported literature method [24-29]. mp: 78–80 °C. ^{1}H NM`R (400 MHz, $CDCl_3$) δ 7.3 (m, 4 H, Ar-H), 6.5 (m, 4 H, Ar-H), 3.7 ppm (s, 4 H, NH_2).

General procedure for the synthesis of organic selenides 4 and 5 via reduction of diselenide 3 and subsequent nucleophilic substitution reaction

Compound **3** (1 mmol) and alkyl halide (methyl iodide or benzyl chloride) (2.2 mmol) were dissolved in EtOH (20 ml). $NaBH_4$ (189.15 mg, 5 mmol) was added portion-wise over 1h. Then

the reaction was stirred for additional 3 hrs. Water was added and the mixture was extracted with dichloromethane. The organic layer was dried and evaporated under a vacuum. The residue was purified by silica gel chromatography.

Synthesis of 4-(methylselanyl)aniline (4)

Compound **4** was prepared from diselenide **3** (344 mg, 1 mmol), methyl iodide (138 µl, 2.2 mmol), and $NaBH_4$ (189.15 mg, 5 mmol). The progress of the product formation was followed by TLC petroleum ether: EtOAc= 6:1, R_f = 0.36, purified by column silica gel chromatography with petroleum ether: EtOAc = 6:1.5. Colorless oil; Yield: 215.55 mg (57%). 4-(Methylselanyl)aniline (**4**) was synthesized as Brown oil according to the reported literature method [30]. ^{1}H NMR (400 MHz, $CDCl_3$) δ 7.31 (d, J = 9.0 Hz, 2 H, Ar-H), 6.61(d, J = 9.0 Hz, 2 H, Ar-H), 3.72 (br s, 2 H, NH_2), 2.66 (s, 3 H, CH_3).

Synthesis of 4-(benzylselanyl)aniline (5)

Compound **5** was prepared from diselenide **3** (344 mg, 1 mmol), benzyl chloride (253 µl, 2.2 mmol), and $NaBH_4$ (189.15 mg, 5 mmol). The progress of the product formation was followed by TLC petroleum ether: EtOAc= 6:1, R_f = 0.36, purified by column silica gel chromatography with petroleum ether: EtOAc = 6:1.5. Colorless oil; Yield: 447.55 mg (85%).^{1}H NMR (300 MHz, $CDCl_3$) δ 7.28 – 7.18 (m, 2H, Ar-H), 7.14 – 7.08 (m, 2H, Ar-H), 6.94 – 6.81 (m, 2H, Ar-H), 6.50 – 6.39 (m, 2H, Ar-H), 3.85 (s, 2H, $SeCH_2$), 3.62 (s, 2H, NH_2); ^{13}C NMR (100 MHz, $CDCl_3$) δ 146.68, 138.65, 137.03, 131.32, 130.48, 120.33, 116.48, 115.63, 32.51; MS (ESI): *m/z* = found 263.85 [M^++1]; calcd. 263.02 [M^+]; HRMS calcd. for $C_{13}H_{13}NSe$ [M^++1]: 264.02860, found 264.02748 [M^++1].

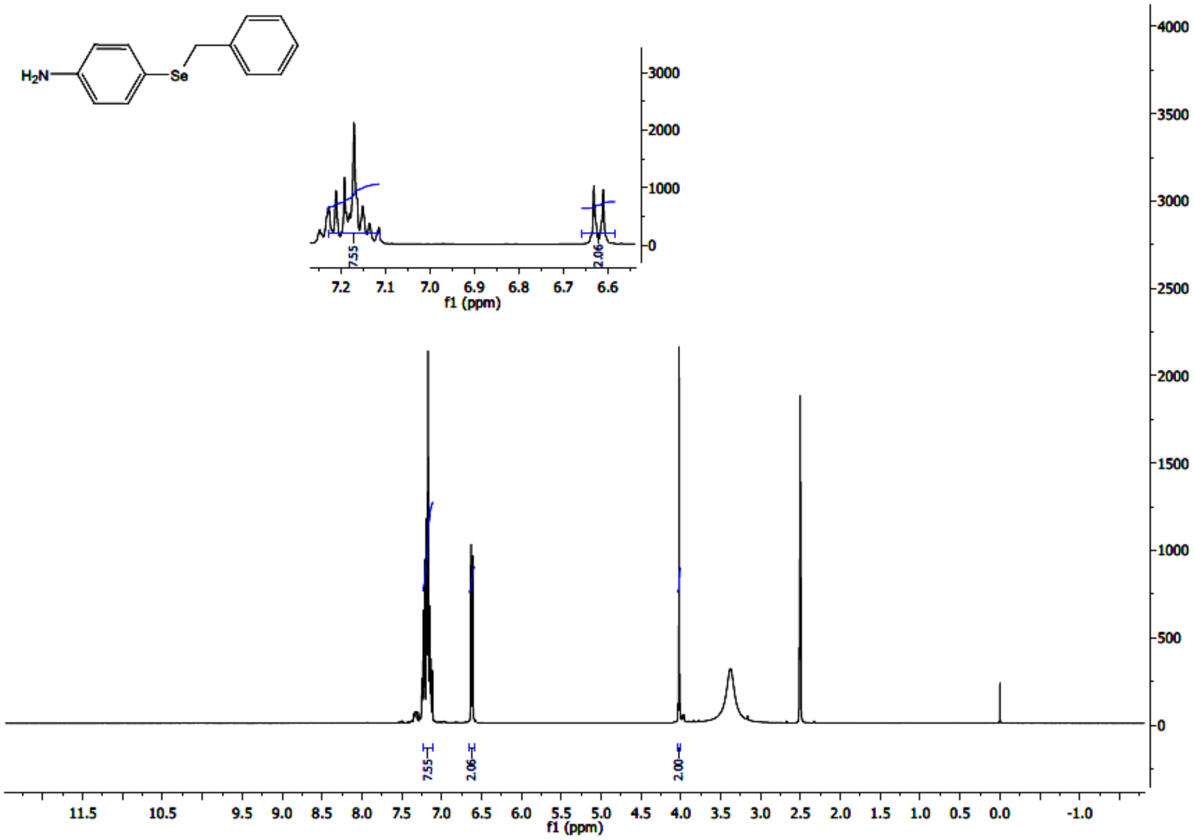

Figure S1. ^{1}HNMR of organoselenium compound 5

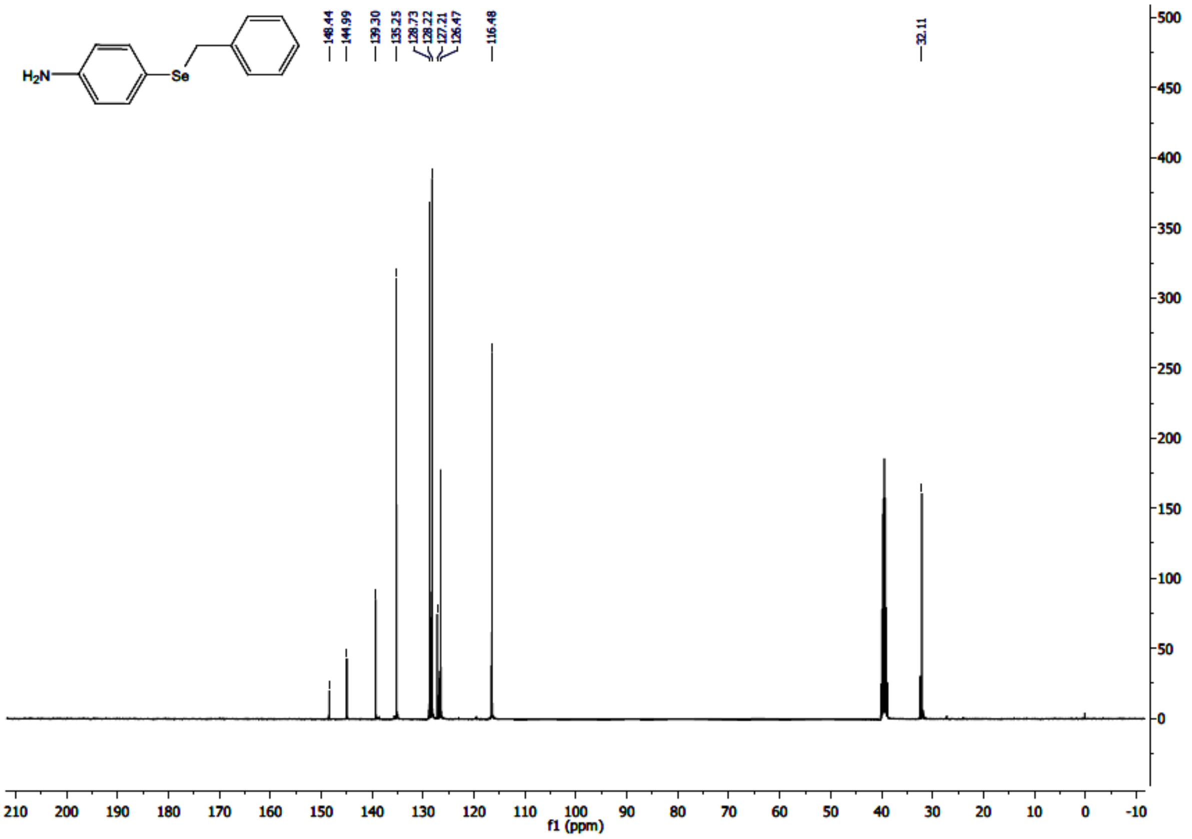

Figure S2. ^{13}CNMR of organoselenium compound 5

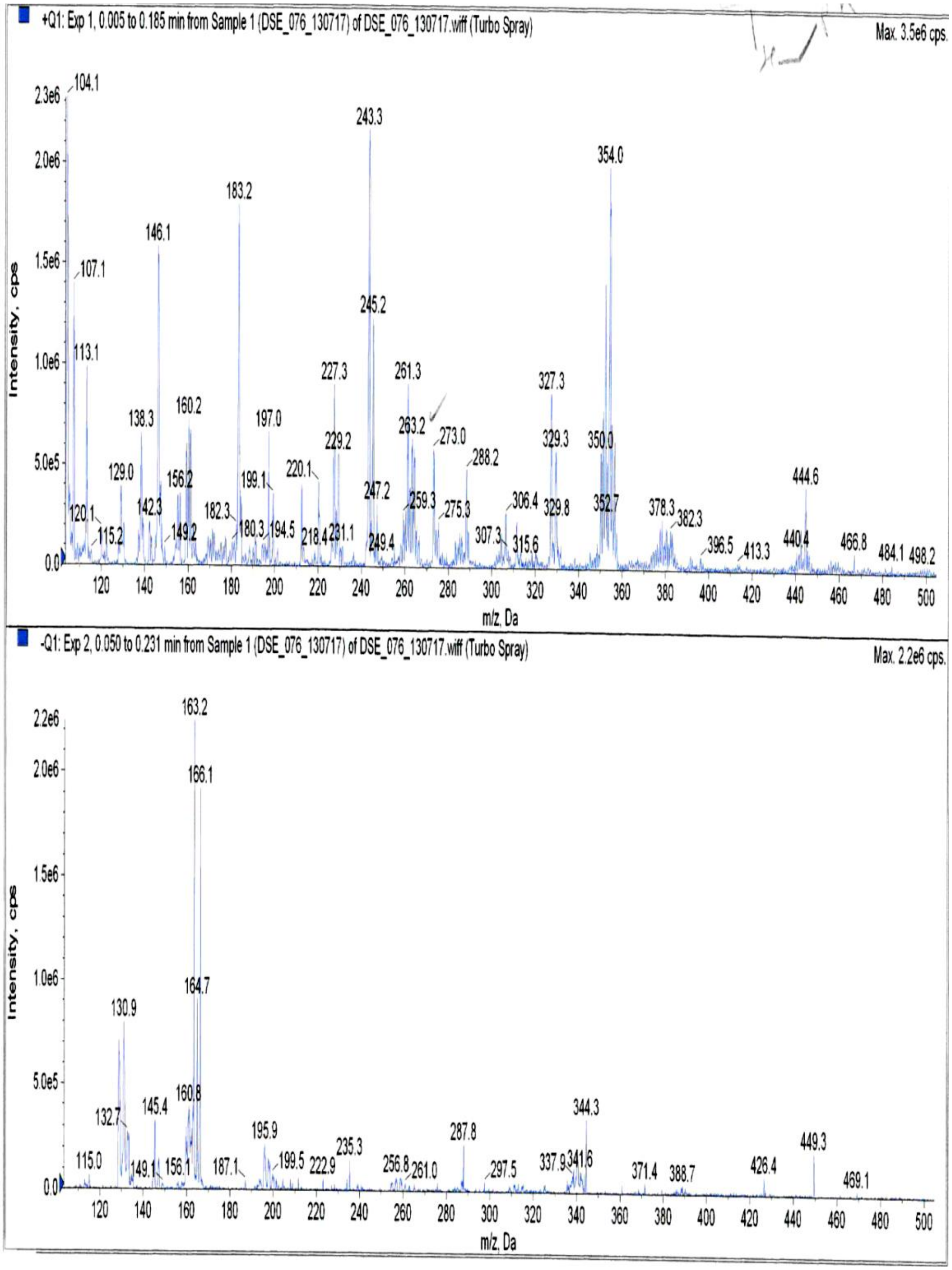

Figure S3. ESI-MS of organoselenium compound 5

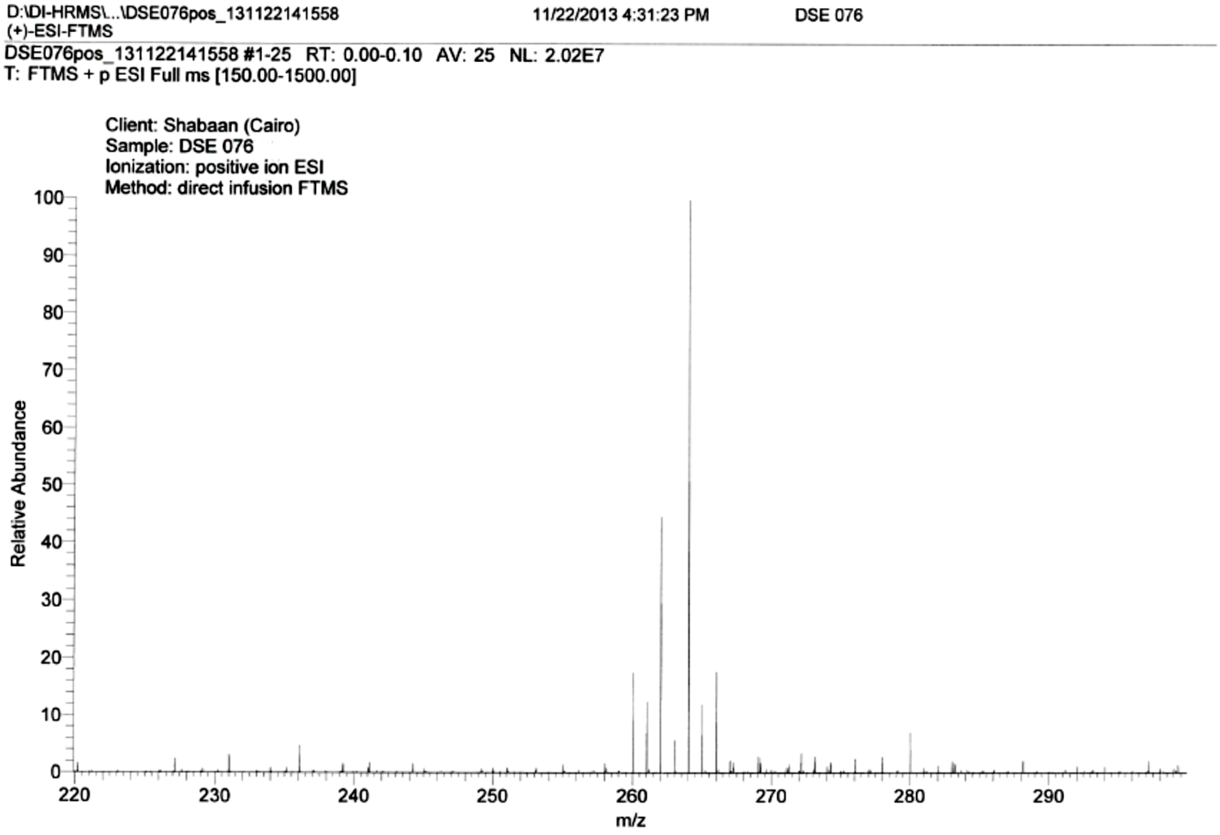

Figure S4. HRMS of organoselenium compound 5

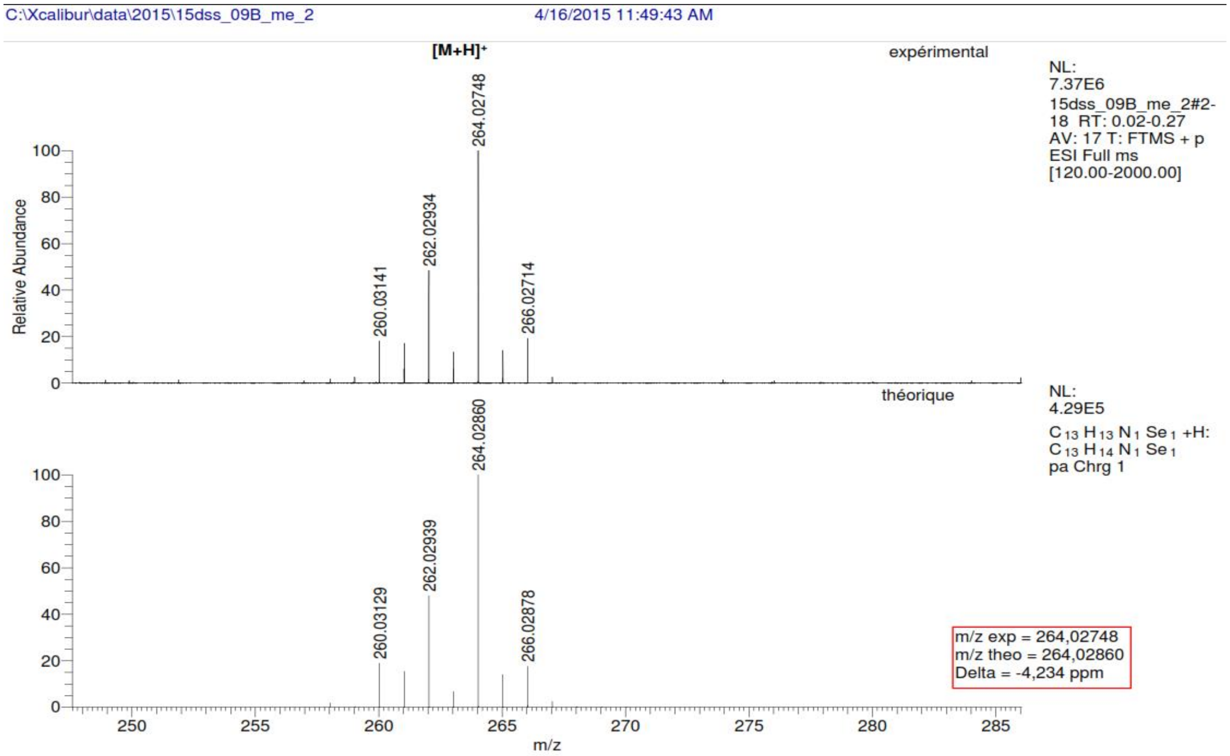

Figure S5. HRMS of organoselenium compound 5

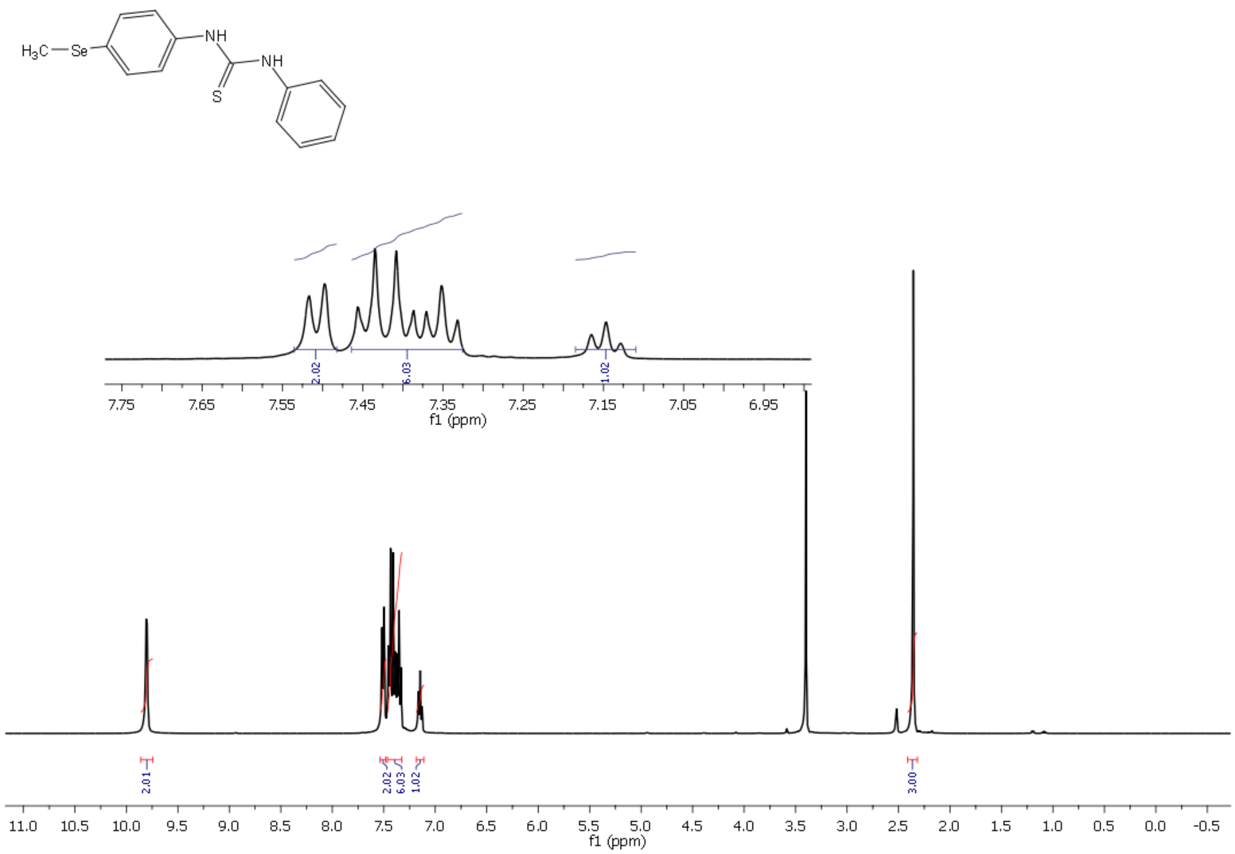

Figure S6. ^{1}HNMR of organoselenium compound DS036

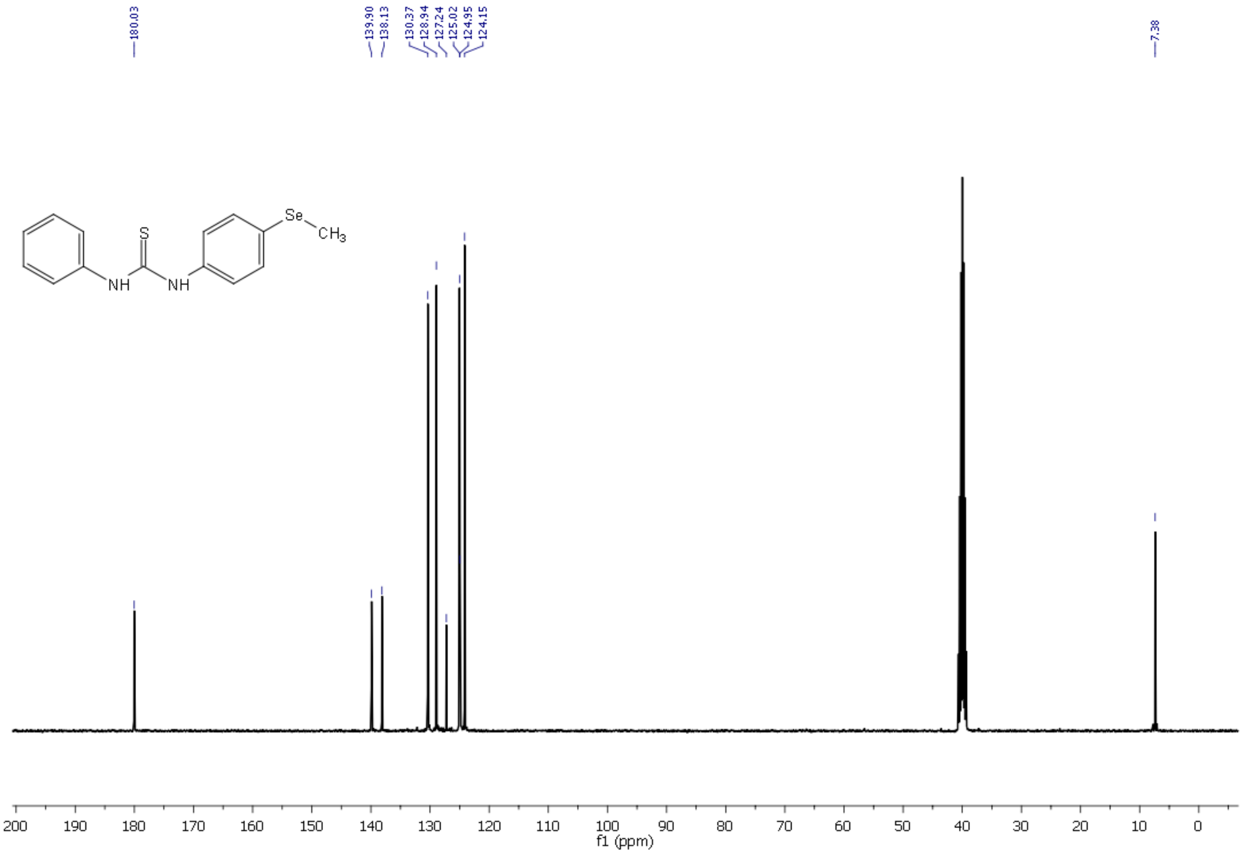

Figure S7. ^{13}CNMR of organoselenium compound DS036

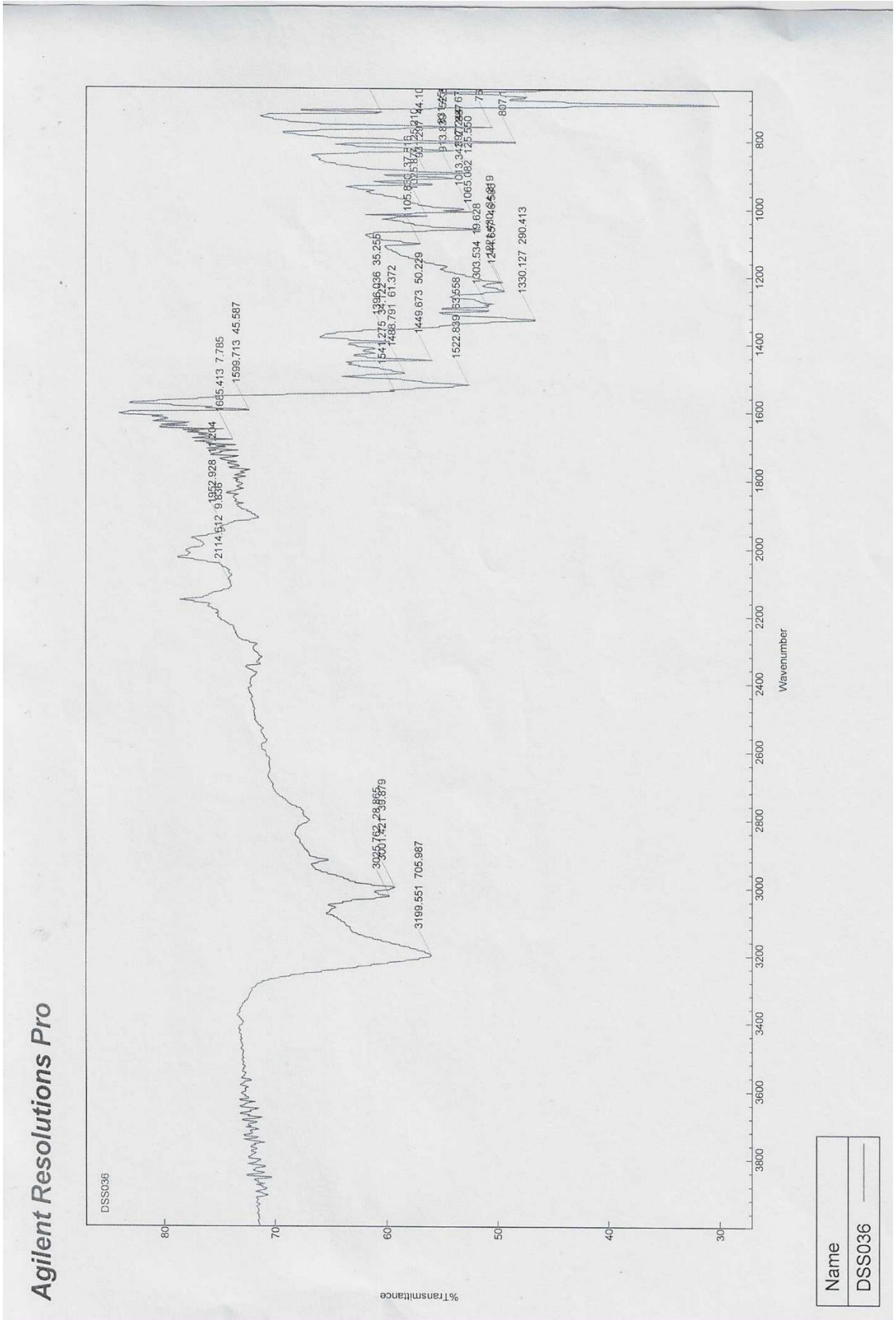

Figure S8. IR of organoselenium compound DS036

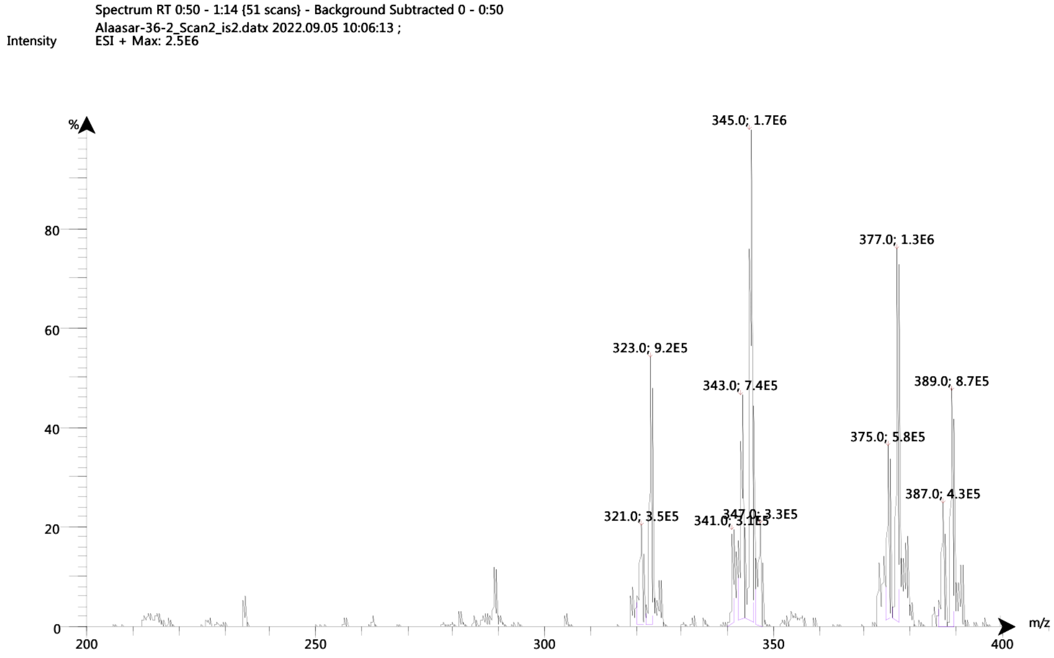

Figure S9. MS of organoselenium compound DS036

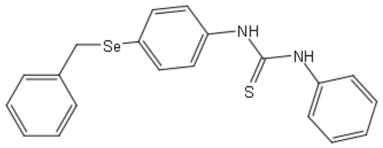

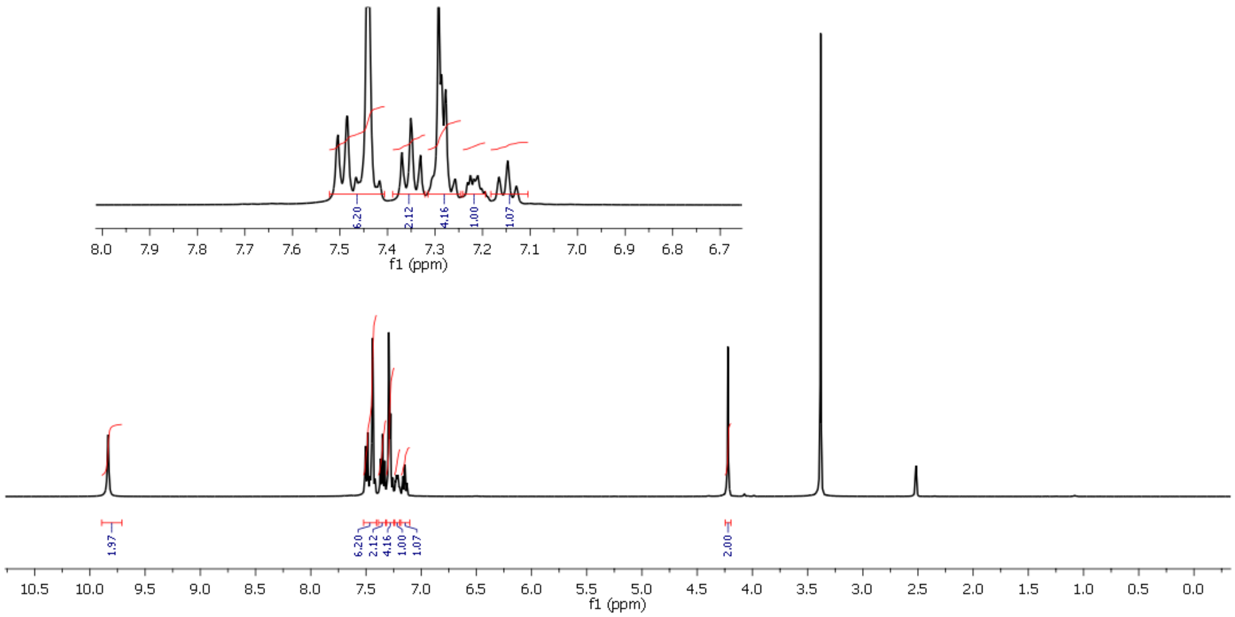

Figure S10. ^{1}HNMR of organoselenium compound DS038

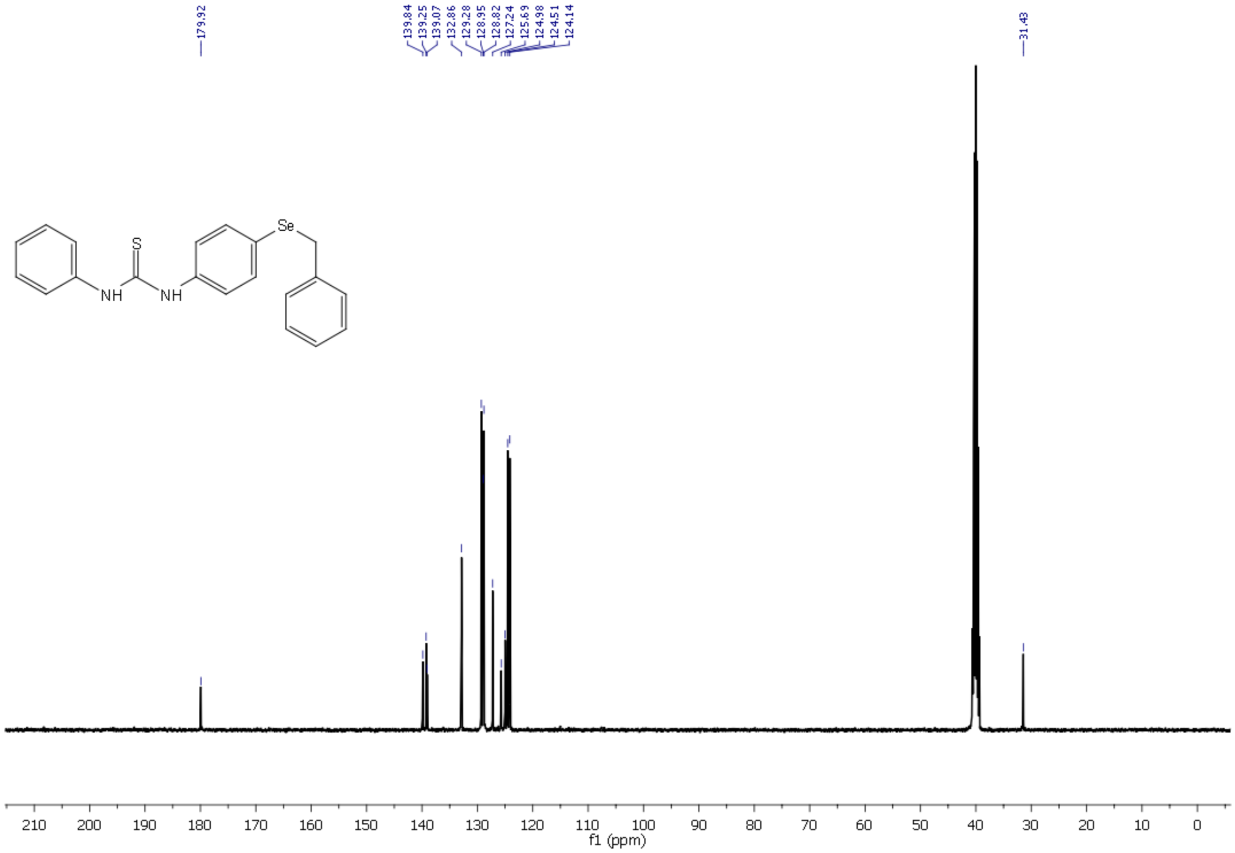

Figure S11. ^{13}CNMR of organoselenium compound DS038

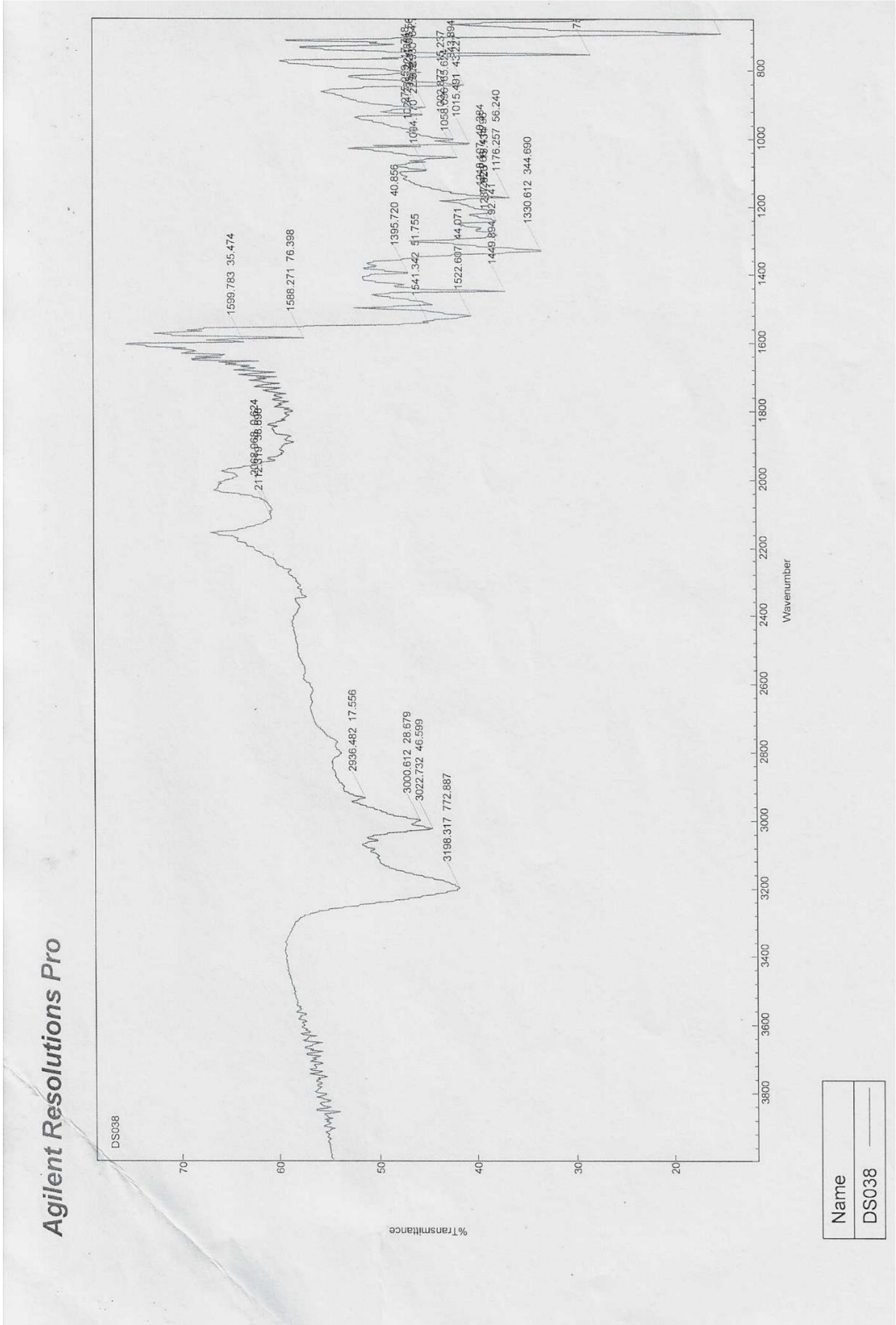

Figure S12. IR of organoselenium compound DS038

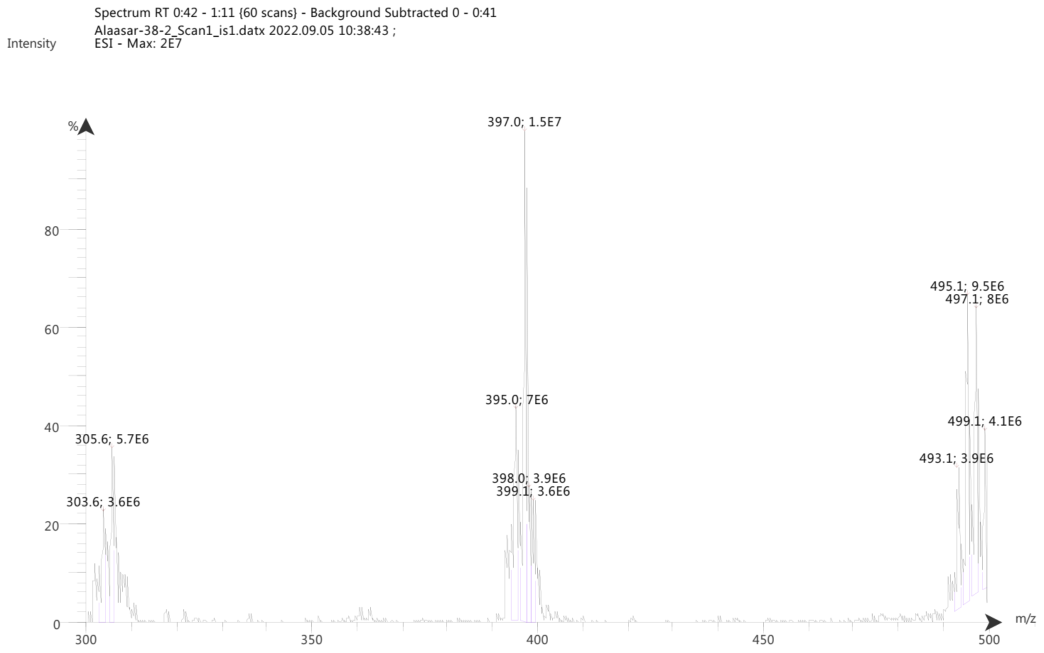

Figure S13. MS of organoselenium compound DS038

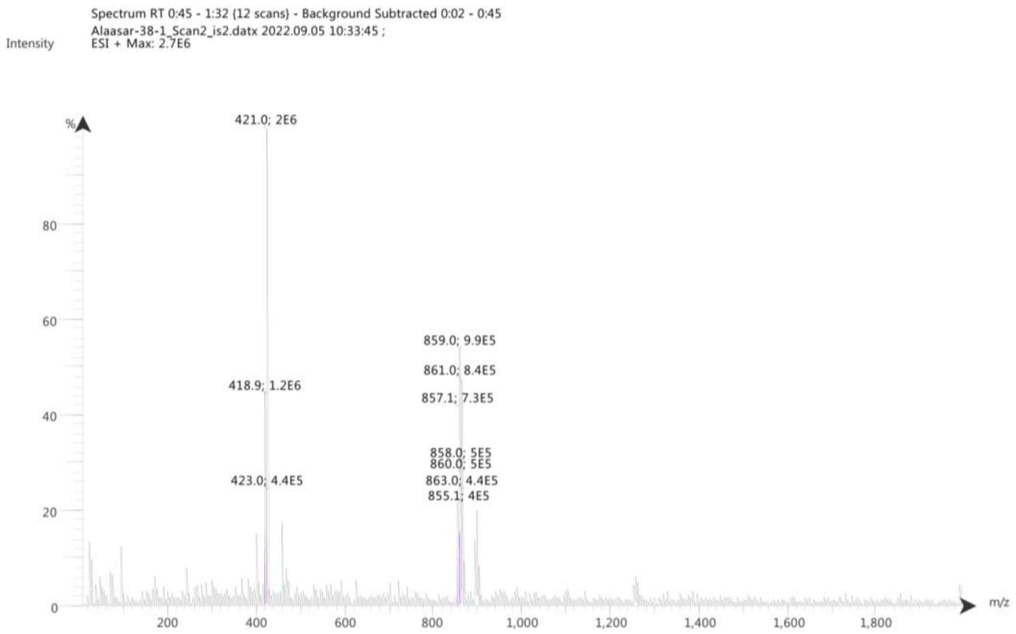

Figure S14. MS of organoselenium compound DS038

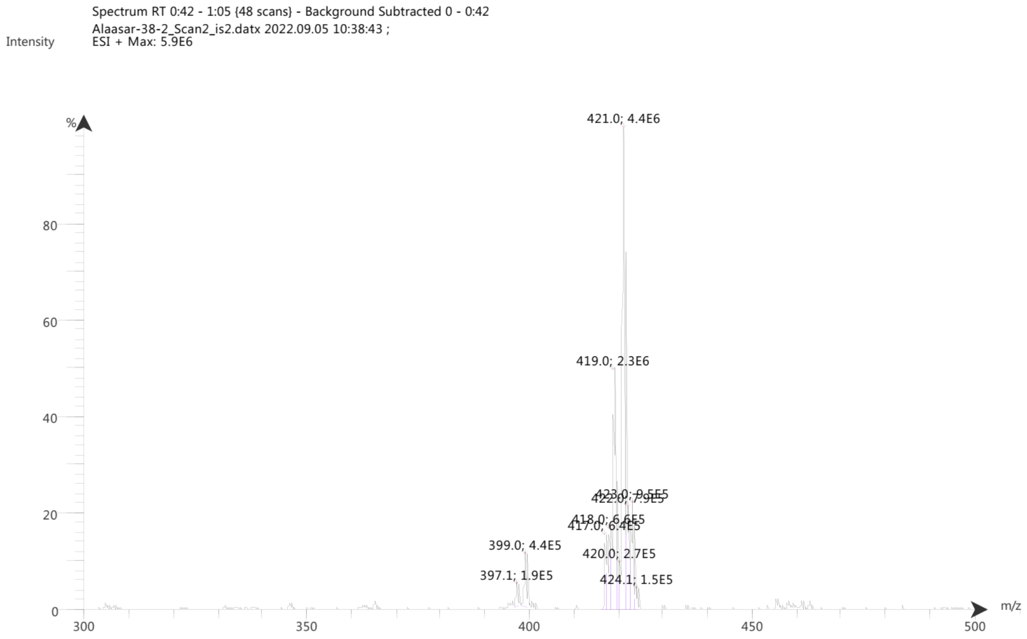

Figure S15. MS of organoselenium compound DS038

Table S1: Adsorption isotherm models and their regression coefficients (R^2) for OSe-based compounds on C-steel in 1.0 M HCl at 298 K

Adsorption isotherms	DS036	DS036
**Langmuir**	0.99998	0.99996
**Frumkin**	0.81451	0.82514
**Temkin**	0.78452	0.81231
**Freundlich**	0.78426	0.87456
